# Multiscale Analysis of Electrochemical Dealloying of Bimetallic Nanoparticles to Tune Catalytic Activity

**DOI:** 10.1021/acsami.6c00288

**Published:** 2026-04-13

**Authors:** Johanna Angona, Dimitrios Valavanis, Daniel Houghton, Fengli Yang, Thais Schroeder Rossi, Jan Clausmeyer, Beatriz Roldan Cuenya, Julie V. Macpherson, Patrick R. Unwin, Kristina Tschulik

**Affiliations:** † Analytical Chemistry, 9142 Ruhr University Bochum, Bochum 44801, Germany; ‡ Department of Chemistry, 2707 University of Warwick, Coventry CV4 7AL, UK; § Hartnoll Centre for Experimental Fuel Technologies, University of Warwick, Coventry CV4 7AL, UK; ∥ Department of Interface Science, 28259 Fritz-Haber Institute of the Max-Planck Society, Berlin 14195, Germany; ⊥ Max-Planck-Institut für Nachhaltige Materialien GmbH, Düsseldorf 40237, Germany

**Keywords:** scanning electrochemical cell microscopy, hydrogen evolution reaction, electrochemical dealloying, bimetallic nanoparticles, scanning transmission electron microscopy, identical-location analysis, reverse micelles, multiscale electrochemical analysis

## Abstract

Electrochemical dealloying is a promising technique to tune the activity and material utilization of electrocatalysts. Studying this process in bimetallic nanoparticles remains challenging since their synthesis yields ensembles with varied properties. To understand the dealloying behavior of individual nanoparticles, we present a combined characterization method that provides statistical distributions in composition, morphology, and catalytic activity. Ag_*x*_Au_*y*_ alloy nanoparticles synthesized via reverse micelles were dealloyed, by potential cycling, to tune their activity for the hydrogen evolution reaction. In a multiscale approach, we performed macroscale measurements on a glassy carbon electrode and scanning electrochemical cell microscopy (SECCM) on small groups of particles/aggregates within the confines of the scanned droplet. The SECCM pipet was used to create different states of dealloying within a single sample, followed by higher-resolution SECCM activity mapping. The faster mass transport in the SECCM tip, compared to the macroelectrode configuration, led to accelerated Ag dissolution from the Ag_*x*_Au_*y*_ nanoparticles. While the particles showed the highest activity and largest Ag content decrease after only 0.5 cycles, heterogeneous behavior was still evident among individual particle groups. Further, using a boron-doped diamond electrode, identical-location scanning transmission electron microscopy and energy-dispersive X-ray spectroscopy studies allowed Ag leaching and particle shrinking to be followed at the single-particle level, as a function of cycle number, for a statistically relevant sample number (*n* > 70). Increased cycling induced particle deactivation, shrinking, and coalescence. Overall, our results showcase an efficient and versatile route to combine systematic tuning and high-throughput screening of nanocatalyst property–activity relationships.

## Graphical abstract

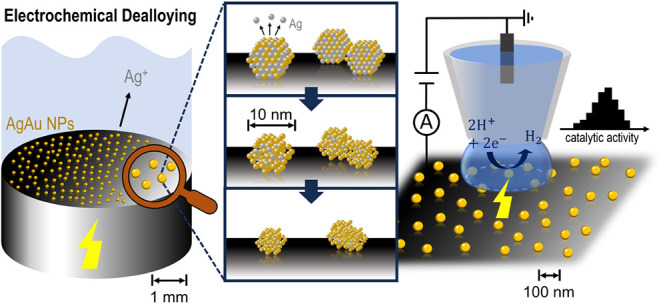

## Introduction

To further advance the hydrogen economy, developing efficient electrocatalysts for water splitting and other electrocatalytic conversions remains a crucial task. Electrochemical dealloying, the controlled dissolution of a less noble metal from an alloy, can be used to tune composition, surface area, and porosity of the electrocatalysts.^1–3^ These parameters have a strong impact on the catalytic activity, as a higher surface-to-volume ratio results in higher current density and improved material utilization.^4–9^ A porous structure can furthermore lead to an increase in low-coordinated surface atoms and a confinement of the reactants in the pores, which can promote catalysis.^9,10^ While platinum group metal-based nanoparticles have the best specific activity,^11^ electrochemical dealloying of nanoparticles of other metals as a method to tune catalytic activity is attracting considerable interest.^3,5,12–14^ As synthesis techniques for multicomponent alloy nanoparticles are further developed, an increasing variety of precursor materials is available.^15–17^ One promising new synthesis method is the potential-induced nucleation of the precursor salts under confinement in reverse micelles.^18,19^

Electrochemical dealloying has previously been shown to have a positive effect on the catalytic activity of nanoparticles beyond surface area enhancement.^14,20,21^ For instance, by potentiodynamic dealloying of Ag_80_Au_20_ nanoparticles, Rurainsky et al. reached a ∼50 times higher catalytic current density at −0.3 V vs the reversible hydrogen electrode (RHE) for the hydrogen evolution reaction (HER).^5^ The enhancement of the surface area-normalized current is often attributed to the formation of low-coordinated active sites^14^ or an induced strain in the nanoparticle crystal lattice.^21^

Understanding the processes that govern electrochemical dealloying is crucial for achieving maximum catalytic activity. Dealloying to form nanoporous gold from planar Ag_*x*_Au_*y*_ electrodes, is relatively well-studied, both experimentally and by simulations.^22–25^ The Ag dissolution rate was found to follow Butler–Volmer kinetics at high overpotential beyond the so-called “critical potential” for dealloying.^2,25^ In order to form a porous electrode, percolation dealloying is required, where the less noble metal can dissolve from both the bulk and the surface. This requires the alloy to contain ≥60 at. % of the less noble metal.^2,26,27^

In the case of nanoparticles, the dealloying processes are generally more complex. According to the few theoretical studies that exist to date, the critical dealloying potential is size-dependent, with higher potentials needed to achieve percolation dealloying at smaller particles.^3,28^ However, below a particle diameter of 15–25 nm no pores have been observed to form.^3,9,12^ Instead, when applying a constant potential, small nanoparticles tend to experience superficial dealloying, resulting in a core–shell structure.^3,7,12^

Given that bimetallic nanoparticle batches used for dealloying are usually polydisperse in size and composition,^16,29^ potentiodynamic dealloying is often chosen, where a varying potential is applied to the electrode to ensure all particles reach their critical potential.^28^ The repeated oxidation and reduction during cyclic voltammetry (CV) further amplifies compositional transformations on the surface, promoting rapid leaching of the less noble metal, percolation dealloying, and porosity formation.^7,9,12^

Despite utilizing a potentiodynamic approach, the dealloying of nanoparticles is often still heterogeneous: some particles may retain much higher amounts of the less noble metal than others, even after the application of many (e.g., 500) cycles.^5^ Besides the effect of a varied particle size, another possible explanation is the different ohmic contact achieved between nanoparticles and the electrode surface.^5^ An immobilization method that ensures good electrical contact is therefore crucial. An additional contribution to the heterogeneous dealloying may be mass transport of the less noble metal ions away from the electrode, which is strongly affected by the particle size and their local packing density on the electrode.^30^ An increased mass transport may therefore help to promote metal dissolution. This was demonstrated in previous work, where increasing mass transport through the application of a magnetic field resulted in more evenly dealloyed and porous particles.^31^ However, this method requires an additional energy consumption in the use of an (electro)­magnet.

Finally, the high surface-area-to-volume ratio compared to bulk materials can also help promote degradation processes during dealloying due to repeated surface rearrangement.^32^ Extended cycling has shown to lead to a coarsening of the nanoporous structure,^32^ dissolution of the more noble metal,^4^ and particle agglomeration,^5^ usually correlated with a drop in the catalytic activity of these particles.^4,5,32^

Since dealloying of nanomaterials is a complex process, dependent on many factors, the macroscopic study of nanoparticle ensembles may only offer limited information. In contrast, microscale dealloying, which directly interrogates a small subset of nanoparticles on the surface, combined with a statistical interpretation, can provide precise insights into structure–activity relationships. To achieve this, scanning electrochemical cell microscopy (SECCM) is employed.

SECCM is a powerful technique that can probe electrochemical properties locally, by the use of a droplet cell formed between a scanned pipet (reservoir) and the working electrode (WE) surface.^33,34^ Repeated landing and retraction of the droplet, in a hopping manner, produces an image of the electrochemical response and topography, with individual interrogation (landing) spots typically having dimensions commensurate to the pipet aperture. This versatile technique has already been employed to investigate electrocatalysis in a multitude of different experiments, ranging from metal grains,^35,36^ grain boundaries,^37,38^ and step edges,^39^ to catalytic investigations of deposited particles,^40–43^ platelets,^44^ etc. on an inert substrate. Depending on the diameter of the pipet tip and the size and dispersion of the particles deposited on the working electrode, the scale of SECCM experiments can range from ensemble measurements, to capturing a few nanoparticles, and even to mapping single particles.^34,40–43^ Combining SECCM with colocated electron microscopy and/or spectroscopic techniques enables the correlation of structural and electrochemical information at the submicron scale.^40,42,45,46^ More generally, recent developments in identical-location electron microscopy techniques have pushed research on nanocatalyst materials to a higher level of resolution, revealing previously inaccessible insights into surface reorganization, facet formation, or degradation of the catalyst or its support.^47–49^

In this work, Ag_*x*_Au_*y*_ nanoparticles electrosynthesized from reverse micelles are studied, using a multiscale workflow and statistical analysis, to reveal their intricate response to electrochemical dealloying and the resulting catalytic activity distributions. First, the primary particles and aggregates, which we collectively refer to as “Ag_*x*_Au_*y*_ entities”, are successively dealloyed at carbon (support) electrodes,^50^ due to their electrocatalytic inactivity compared to metal electrodes, and their HER activity is assessed, using linear sweep voltammetry . Then SECCM is used as an accelerated testing platform to gain statistical distributions for the particles’ specific activities and compositions on the nanoscale.

For that, the dealloying is done locally by approaching a large SECCM pipet to several locations on the support electrode and varying the number of dealloying cycles. This is followed by a second colocated SECCM experiment with a smaller probe size to analyze the catalytic activity and Ag content of small groups of Ag_*x*_Au_*y*_ entities (typically 5–10) at different dealloying states.

Finally, the structural and compositional responses of the particles to the potentiodynamic dealloying are studied in detail by identical-location scanning transmission electron microscopy (IL-STEM) and energy-dispersive X-ray spectroscopy (EDX) analysis, using an electron beam transparent carbon electrode.^51^ In this way it is possible to make identical-location measurements at a statistically relevant number of Ag_*x*_Au_*y*_ entities at different states of dealloying. While we use Ag_*x*_Au_*y*_ nanoparticles with an average composition of *x* = 60 and *y* = 40 as a model precursor, this efficient and rigorous multiparameter characterization may be applied to other systems, to assist in the tailored optimization of bi- and multimetallic nanocatalysts.

## Results and Discussion

### Voltammetry on a Macroelectrode

We first consider the dealloying and resulting electrocatalytic properties at the macroscale. Achieving a homogeneous alloy structure is a challenging task, as bimetallic nanoparticles tend to form a core–shell structure during bottom-up synthesis.^52^ Therefore, Ag_60_Au_40_ nanoparticles were directly generated on a glassy carbon (GC) electrode by potential-induced nucleation of the precursor salts. The precursors HAuCl_4_ and AgNO_3_ were encapsulated in reverse micelles of the block copolymer polystyrene-*b*-poly­(2-vinylpyridine) (PS−P2VP).^18,53,54^ In contact with the biased electrode, these nanoreactors (shown in Figure S1) result in the formation of bimetallic nanoparticles of defined, monomodal size (see Figure S2, average diameter 11 ± 3 nm), and homogeneous alloy structure,^18^ conceptually similar to emulsion-based electrodeposition approaches,^55^ but with superior size control.^19,56^ The use of electrodeposition ensures that every nanoparticle is in electrical contact with the substrate, surpassing a common obstacle in particle batches formed via wet-chemical synthesis and then dropcast on the support electrode.^57^ The composition of ∼60% Ag and ∼40% Au was chosen here to create Ag-enriched particles and ensure long-term stability of the reverse micelles without precipitation of AgCl.

Following the same procedure used previously,^5^ the micelle-derived Ag_60_Au_40_ nanoparticles on the macroelectrode were electrochemically dealloyed by CV, with exemplary polarization curves presented in Figure 1a. The dealloying cycles showed a clear decrease in the height of the oxidation (A) and reduction (C) peaks with cycle number, indicative of Ag dissolution.^5,12^ The first dealloying cycle showed two distinct oxidation peaks which are attributed to the dissolution of differently coordinated Ag atoms in the alloy. First, Ag is dissolved from the nanoparticle surfaces in A1, followed by dissolution of alloyed Ag from the bulk in A2.^12^ In the literature, the second process is often detected as a broad region of increased current rather than a distinct peak.^5,12,31^ This can also be observed here in the second and following cycles. At ∼1.2 V vs Ag|AgCl an onset is measured which was attributed to the oxidation of the Au surface to AuO*_x_*.^12,58,59^ The potential was reversed here to avoid extensive passivation of the Au-rich particles. Dissolution of Au is not expected in this potential range in HClO_4_.^60,61^

**Figure 1:**
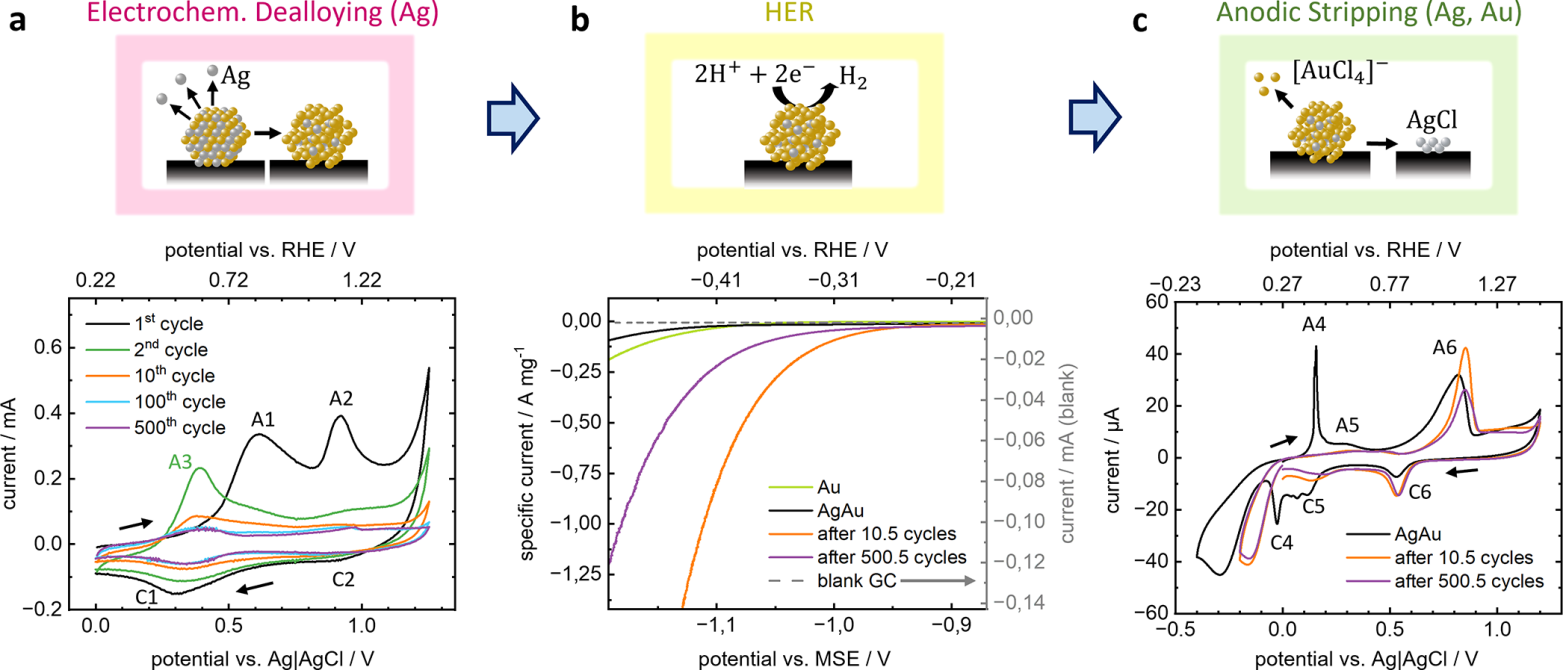
Electrochemical measurements on dealloyed Ag_60_Au_40_ entities on a GC macroelectrode. Schematics of particle modifications/catalysis and corresponding voltammograms. (a) Selected CVs of electrochemical dealloying in 1 M HClO_4_ using a scan rate of 1 V s^–1^ and Ag|AgCl|3 M KCl reference electrode (RE), starting at 0 V vs Ag|AgCl, followed by (b) linear sweep voltammetry in 0.5 M deaerated H_2_SO_4_ at 2 mV s^–1^ scan rate (potential vs mercury-mercurous sulfate reference electrode (MSE, sat.), specific current by mass of nanoparticles, derived from anodic stripping peaks) of micelle-nucleated Ag_60_Au_40_ particles at three states of dealloying, compared to micelle-nucleated Au nanoparticles and absolute current at a blank GC electrode, and (c) anodic stripping of the same nanoparticles as in (a,b) in 0.1 M HCl at 25 mV s^–1^ scan rate and using 0 V vs Ag|AgCl as the starting potential.

As the potential is reversed, the AuO*_x_* is reduced back to Au, which can be detected by a small peak at ∼0.95 V vs Ag|AgCl (C2). In agreement with literature,^5,12^ the dissolved Ag is partially redeposited in peak C1, followed by dissolution in the second cycle (A3). The potential difference between A3 and A1 indicates that a lower overpotential is needed for the dissolution of the redeposited Ag than of Ag atoms from the alloy nanoparticles as the latter ones are partly stabilized by surrounding Au.^5,62^ To assess the catalytic activity of the nanoparticles after performing *n* dealloying cycles, the dealloying was always stopped at the upper anodic potential, yielding *n* + 0.5 cycles, in order to avoid Ag redeposition.

Linear sweep voltammetry was performed in deaerated H_2_SO_4_ solution to assess the electrocatalyst activity for the HER, while excluding any overlap with the oxygen reduction reaction. As shown in Figure 1b, the as-deposited Ag_60_Au_40_ alloy particles exhibited poorer mass activity for HER compared to monometallic Au particles electrodeposited from the same block copolymer reverse micelles. After 10.5 cycles of dealloying treatment, a ∼35 times increase in specific current for HER at −1.1 V vs MSE sat. was detected. After further dealloying by cycling 500.5 times in HClO_4_ the catalytic activity at −1.1 V vs MSE sat. was only ∼27% of the activity achieved after 10.5 cycles. This catalyst deactivation with extended cycling has previously been determined to be related to particle agglomeration and Au passivation.^5^ The IL-STEM analysis performed in this study (see below) provides further insights into the particle morphology.

After measuring the catalytic ensemble activity, the nanoparticles were electrochemically dissolved by anodic CV stripping in acidic chloride solution (HCl). The procedure used herein has been applied to Ag_*x*_Au_*y*_ nanoparticles previously^5,62,63^ to fully oxidize both compounds in one cycle. The anodic stripping CV (Figure 1c) hence provides information about the average composition, type of bimetallic nanoparticles (alloy, core–shell, etc.) and total mass of Ag and Au present on the electrode.^5,62,63^ Anodic stripping CVs were recorded for three new batches of nanoparticles: as-synthesized, as-synthesized subject to 10.5 dealloying cycles and as-synthesized subject to 500.5 dealloying cycles. The black curve of the as-synthesized particles has the typical shape of Ag_*x*_Au_*y*_ alloy stripping.^62,63^ In the presence of Cl^–^, metallic Ag is expected to transform to AgCl following:^62^

1 $$\mathrm{Ag}+{\mathrm{Cl}}^{-}⇌\mathrm{AgCl}+e^{-}$$

The associated transfer of one electron per silver atom is detected in the CV as a sharp anodic peak (A4) at ca. 0.16 V vs Ag|AgCl, indicating dissolution of surface Ag.^62^ The peak is followed by a broad shoulder (A5), indicating the dissolution of alloyed Ag from the bulk.^5,62^ Figure 1c shows that the sharp anodic peak (A4) entirely disappears after 10.5 dealloying cycles while a broader peak (near A5) remains apparent and is shifted to a higher potential. This potential shift indicates that with proceeding dealloying, the particles get more Au-enriched, so that remaining Ag atoms in the bulk are more and more stabilized by the surrounding Au matrix, requiring higher anodic potential for dissolution.^62,64^ In the presence of Cl^–^, Au is dissolved by a mixture of two complexation processes,^65,66^

2 $$\mathrm{Au}+2{\mathrm{Cl}}^{-}⇌{[{\mathrm{AuCl}}_{2}]}^{-}+e^{-}$$

3 $$\mathrm{Au}+4{\mathrm{Cl}}^{-}⇌{[{\mathrm{AuCl}}_{4}]}^{-}+3e^{-}$$

with soluble products. This is observed by peak A6 which shows a slight decrease in peak area from the pristine state to the 500.5 cycles, which may relate to unwanted dissolution of Au during dealloying. After peak A6, the current stays at a value above the capacitive baseline, indicating that Au dissolution and oxidation are in competition at these potentials.^45^ Figure 1b and c indicates that after 10.5 cycles of dealloying, most of the Ag has been dissolved, resulting in a large increase in catalytic current.

In this work, we used the mass-normalized current density (not the surface area normalized current density) as a measure of specific activity in order to also include the impact of morphological changes (surface-area-to-volume ratio) during dealloying. Therefore, the observed increase in activity may also be related to an increase in specific surface area. The mass of the nanoparticles was calculated from the charge of the stripping peaks depicted in Figure 1c following the method described in S2.1, Supporting Information. A comparison between normalization by mass and by surface area, however, showed the same quantitative trend of catalytic activity with a maximum after 10.5 dealloying cycles (see S2.2).

The macroscale experiments conducted here yield the sum of the responses of all nanoparticles present on the macroelectrode to the different dealloying treatments. However, our questions regarding the statistical variations between the particles remain unanswered, e.g.: How homogeneous is the particle composition distributed? How uniform is the individual Ag content of the particles reduced by dealloying? Do all particles contribute equally to the enhanced activity after dealloying, or is this dominated by a few nanoparticles with exceptionally high specific activity?

### Combined SECCM Experiment

In order to answer the questions posed above, submicroscale electrochemical measurements were performed using SECCM, which can provide detailed insights into the nanoparticles’ heterogeneous behavior under electrochemical dealloying.

We demonstrate a two-step workflow to dealloy deposited nanoparticles on a GC electrode to different extents, and then rapidly screen their Ag content and specific activity toward the HER.

First, using a larger pipet tip (65 μm aperture diameter), the Ag_60_Au_40_ nanoparticles were locally modified using four different electrochemical dealloying treatments, in four different areas of the electrode surface (Figure 2a–b). Then, a smaller probe (400 nm diameter) was used in an Ar environment to screen the specific activity and Ag content of the nanoparticles in varying dealloying states using CV (Figure 2c–d). Across each area, multiple SECCM measurements were recorded (>20 in each area of different dealloying treatments, ∼400 in the surrounding area of as-synthesized nanoparticles). Using this multiscale workflow, we show that SECCM can be used as an accelerated testing platform with high throughput, helping to identify optimal conditions for the particle treatment.

**Figure 2:**
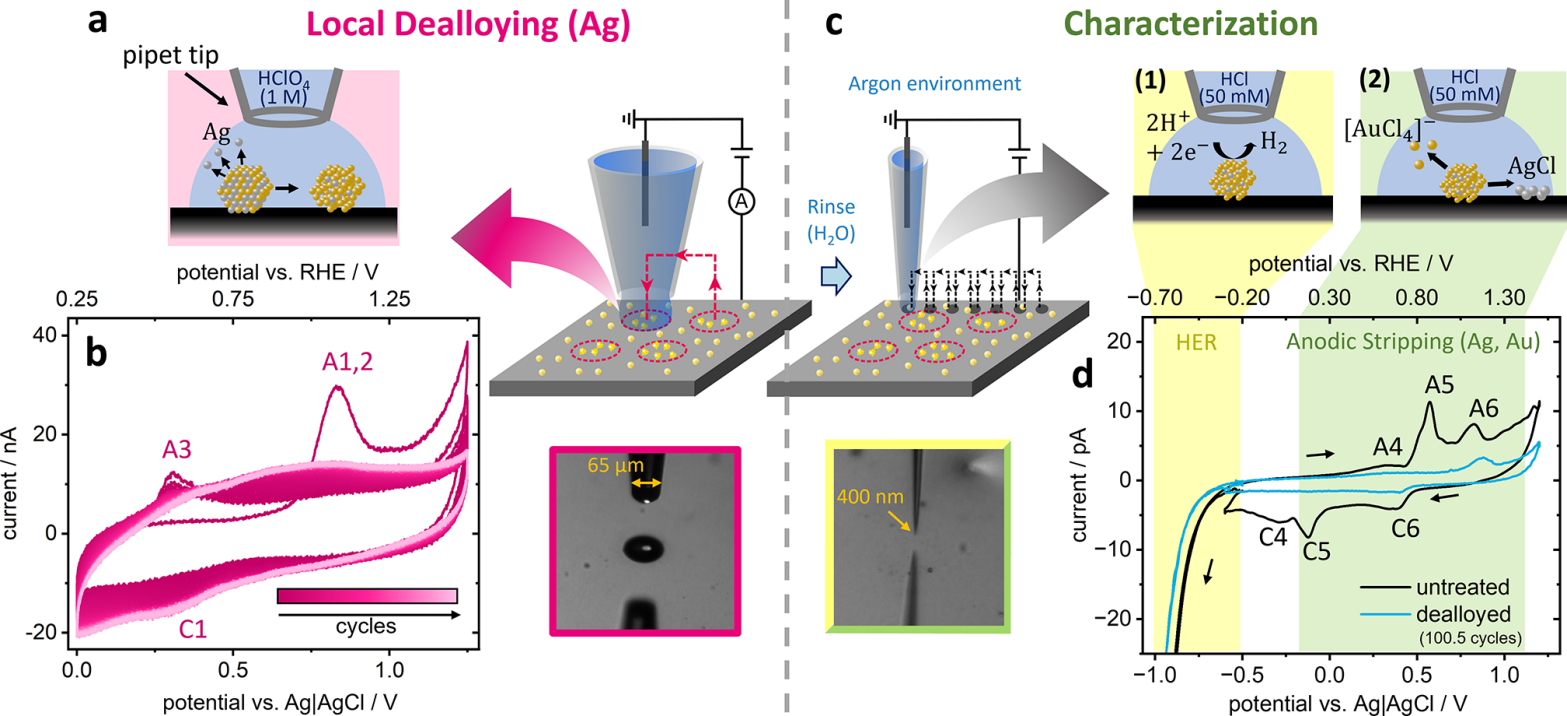
Catalyst tuning and characterization starting with (a) local dealloying in four different spots on a GC electrode with a 65 μm diameter HClO_4_-filled tip using different numbers of CV cycles (i.e., different final dealloyed states), (b) CV example of 100.5 cycles in 1.0 M HClO_4_ at 1 V s^–1^, starting at 0 V vs Ag|AgCl, (c) after gently rinsing the sample with ultrapure water, a 400 nm diameter HCl-filled tip was used under an Ar atmosphere for characterization, (d) exemplary CVs on few untreated and dealloyed nanoparticles (at 100.5 cycles) at 0.5 V s^–1^ in 50 mM HCl starting with HER (3 cycles between −0.5 V and −1 V) followed by anodic stripping and redeposition of Ag and Au (1 cycle from −0.5 V over 1.2 V to −0.6 V).

The electrosynthesis from reverse micelles yielded a high coverage (∼10 Ag_60_Au_40_ entities per μm^2^) on the GC electrode (see Figure S4), leading to many nanoparticles exposed to the electrochemical dealloying treatment in each of the four approaches with the 65 μm diameter micropipet. Shorter dealloying cycle treatments of 0.5, 1.5, 10.5, and 100.5 cycles in 1.0 M HClO_4_ were chosen here to gain more precise insights into the initial dealloying cycles during which the strongest change in activity was found (Figure 1a). All of the 100.5 dealloying cycles in one of the landings with the large pipet tip are presented in Figure 2b. The shape of the CVs was found to be reproducible in all four spots of dealloying. In contrast to the dealloying CVs on the macroelectrode (Figure 1a), we only observe one prominent peak in the first cycle during dealloying with the micropipet, which may be related to a mixture of Ag dissolution from the alloy and Au oxidation (A1,2). Ag redeposition and dissolution from the surface can be observed by peak C1 and A3, respectively. While peak A3 is decreasing with cycle number, a plateau is forming around peak A1,2 at higher cycle numbers, indicating a shift in the Ag dissolution potential as less Ag is dissolved from the surface and more Ag is dissolved from the bulk.^5,31,62^

After dealloying, a subsequent SECCM experiment with a 400 nm diameter pipet tip was aimed at screening the catalytic activity as a function of the number of dealloying cycles. Furthermore, SECCM was used as an analytical tool to estimate the composition of the material confined in the droplet cell. To achieve this, the two experiments on the macroelectrode presented in Figure 1b–c (catalytic measurement plus anodic stripping) were combined in a single SECCM mapping experiment on the submicron scale. Consequently, the obtained catalytic activity was measured in HCl here to connect the anodic stripping experiment directly to the measurement. The electrolyte concentration was downscaled for the SECCM experiment, in order to avoid precipitation at the tip. Therefore, a direct comparison between the activity values obtained with SECCM and the ensemble activity on the macroelectrode is not feasible.

Comparative scanning electron microscopy (SEM) imaging (S3.2, Supporting Information) revealed that ∼5–10 Ag_*x*_Au_*y*_ entities were likely to have been probed at each landing point of the HCl-filled pipet. Figure 2d presents two exemplary CVs of HER characterization and anodic stripping in 50 mM HCl on untreated and 100.5-cycle dealloyed Ag_*x*_Au_*y*_ entities. Starting with 3 cathodic cycles (of which only one is presented in the figure), the catalytic activity was assessed first. Then the potential was swept anodically to (partially) dissolve the particles, obtaining stripping peaks for Ag and Au, for compositional analysis.

Similar to the example in Figure 2d, clear dissolution peaks were found in all the CV curves in the SECCM experiment, confirming that at least one particle was measured in each location. Notably, the peak positions and heights in the anodic stripping CVs of the as-deposited particles differ from those obtained on the macroelectrode (Figure 1c). Specifically, the potential of peaks A4 and A5, at which Ag is oxidized in the presence of chloride, under one electron transfer according to eq 1, was more positive in the case of SECCM than in the macroscopic measurement. Moreover, while peak A4, related to the dissolution of lower coordinated Ag, is much smaller here; peak A5, which is attributed to the dissolution of higher coordinated Ag, is more pronounced. This shows that the same type of particles can yield differently shaped current transients depending on whether they are probed within a large ensemble on a macroelectrode or in small particle groups—or individually—in the SECCM droplet cell.

The differences in the electrolyte concentration and scan rate used in SECCM compared to the macroelectrode experiment should not be ignored. However, direct comparisons of the current transients within the SECCM data set are possible. It is noted that in the stripping CVs of dealloyed vs as-synthesized particles (Figure 2d), peaks A4 and A5 are greatly diminished, while peak A6 is almost unaffected by dealloying—confirming that the first two must be related to Ag dissolution, while the latter can be attributed to the stripping of Au.

Figure 2d also shows that the current overall decreased in the dealloyed areas compared to the as-synthesized areas. Consequently, the absolute HER current was found to be lower on the dealloyed particles. This lower current gives rise to the question of whether some particles may have been dissolved or detached from the electrode as a result of dealloying, leading to a significant material loss. This undesirable particle decay could have been exacerbated in the SECCM by the increased current density due to the much smaller active (wetted) area, resulting in electrochemically harsh conditions. However, SEM imaging of the measured areas after SECCM revealed no extensive change in the particle coverage (Figure S4). Therefore, the question remains open as to whether the particles undergo any morphological changes during dealloying. This is discussed further in the section detailing IL-STEM analysis (next section).

Analogously to the analysis on the macroelectrode, we consider the charge of the anodic stripping peaks as a measure for the transferred mass of Ag and Au, in order to normalize the HER current and determine the Ag content. Again, due to the lower electrolyte concentration and higher scan rates employed compared to the macroelectrode experiment,^62,63^ the quantities obtained for Ag content and nanoparticle mass are handled as relative quantities and only used to make comparisons between different SECCM data sets.

For the analysis, the dissolution peaks of all CVs obtained in the SECCM experiment with the 400 nm diameter tip were integrated, after manual background subtraction, due to the high background current and possible overlap between peaks A5 and A6. The Ag content was determined from the peak charge by means of Faraday’s law, assuming one electron transfer per Ag to AgCl transformation. For the Au peak, a mixture of reactions shown in eqs 2 and 3 was assumed with an average electron transfer of 1.9 electrons per Au atom as found in previous works.^65,66^ The number of Ag atoms divided by the sum of Ag and Au atoms yields the Ag content (for calculation see S2.1, Supporting Information).

Figure 3a shows the computed Ag content using the 400 nm pipet SECCM tip, for every pixel (20 μm × 20 μm) representing an individual, submicron scaled, experiment within the droplet cell. The hopping distance between the individual locations was 20 μm while the droplet diameter was <1 μm, meaning only a fraction of the modified area was covered by the SECCM probe. The clear difference in Ag content with respect to the surrounding untreated areas confirms successful dealloying in the four areas wetted during the first (larger probe diameter) SECCM experiment. The sizes of the four dealloyed spots in Figure 3a appear to depend on the number of dealloying cycles, indicating a spreading of the droplet over time. This is confirmed by the increasing background current with cycle number for the 100.5 dealloying CVs in Figure 2b.

**Figure 3:**
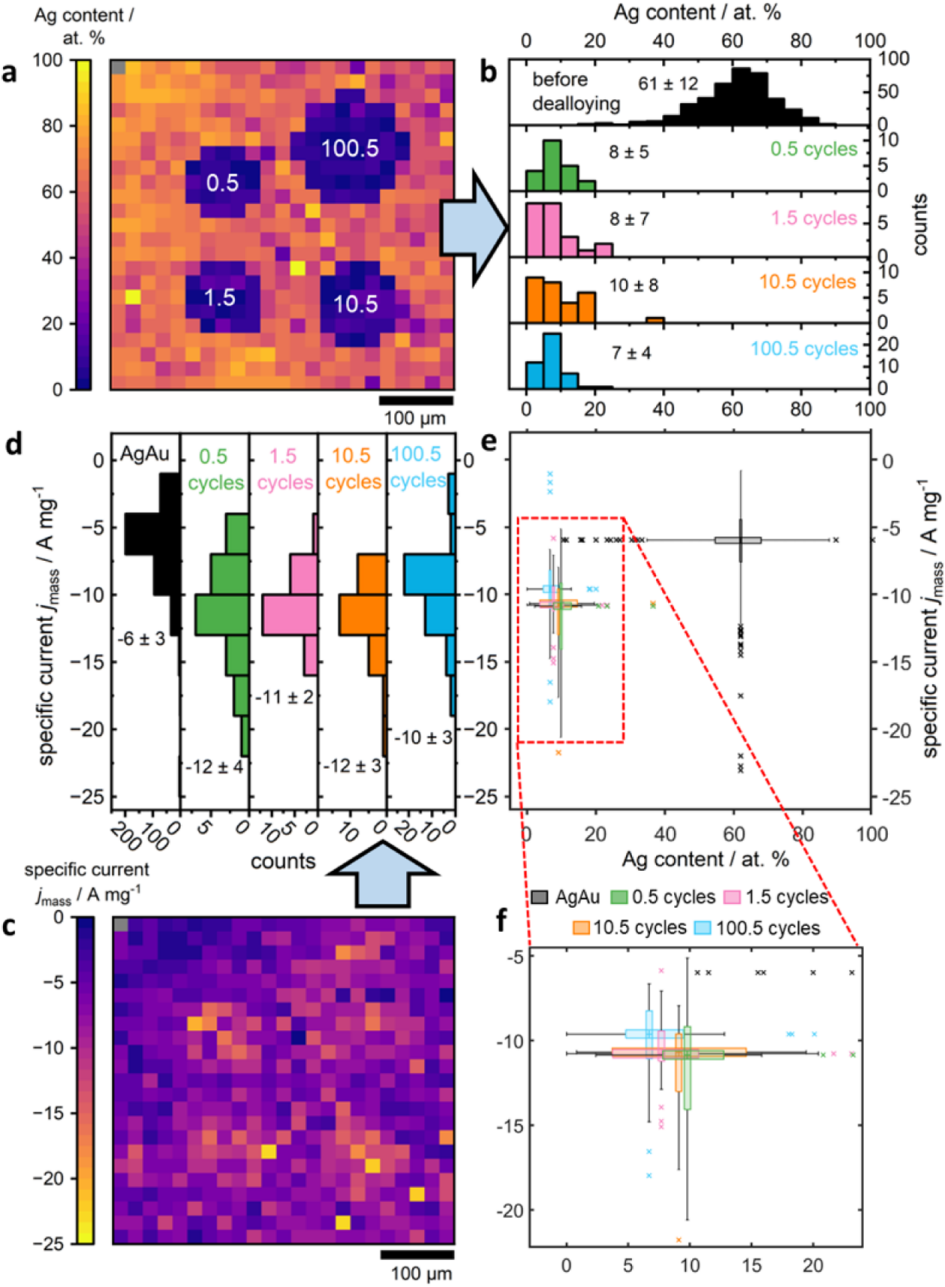
Characterization of dealloyed Ag*_x_* Au*_y_* entities by SECCM with 400 nm diameter pipet, using 50 mM HCl. (a) SECCM map of Ag content across a GC/entity surface comprising of 4 dealloyed spots (SECCM tip diameter = 65 μm) and surrounding untreated areas, derived from integration of anodic stripping peaks; number of dealloying cycles in white, (b) Ag content histograms from the SECCM map in (a), (c) SECCM map of mass-related current density *j*_mass_ at −0.65 V vs RHE of the third CV cycle in HER potential range, (d) *j*_mass_ histograms from the SECCM map in (c), (e) 2D box charts for correlation of Ag content and catalytic activity distributions from SECCM where the medians of both properties were overlaid, and outliers were computed using the interquartile range, (f) zoom-in into (e) at the distributions from dealloyed particles. The underlying data is additionally available in Table S2.

Choosing a different representation of the data in Figure 3a, b comprises the compositional distributions of the individual particle groups as histograms. It is noteworthy that the Ag content was already reduced to an average of 8% ± 5% after the first 0.5 cycles, while no further significant Ag loss could be observed with further cycling. The particle composition was confirmed by random point-EDX measurements post-SECCM (Figure S5). The results indicate that the Ag content may have been slightly underestimated by the peak-integration in SECCM, possibly due to the overlap between peaks A5 and A6.

Similar to calculating the relative Ag content, we determined the mass to normalize the catalytic current, in every location of the SECCM experiment, by integrating the Ag and Au dissolution peaks (see S2.1). As a measure for the catalytic activity toward HER, the current was taken from the third CV cycle at −0.65 V vs RHE, to ensure equilibration of the droplet–electrode interface.

Only a small variation between the three polarization curves was detected, indicating good droplet stability. After normalization, the resulting mass-related specific current *j*_mass_, on as-synthesized and dealloyed nanoparticles at the four dealloying states, is represented in Figure 3c–d. The statistical distributions of catalytic activity and relative Ag content were correlated using 2-dimensional box charts presented in Figure 3e–f, where the medians of both distributions were overlapped for each number of dealloying cycles. The upper and lower limits of each box represent the upper and lower quartile, while outliers (marked with an “x”) are defined as values that are more than 1.5 times the interquartile range away from the upper or lower limit of the box. The ends of the whiskers mark the maximum and minimum values of the distribution that were not classified as outliers.

The four brighter areas in Figure 3c and the histograms in Figure 3d confirm that the specific activity of the particles was enhanced by dealloying, with the most prominent change happening during the first 0.5 cycles (absolute increase from −6 ± 3 A mg^–1^ to −12 ± 4 A mg^–1^). The variability between the catalytic activity of different groups of Ag_*x*_Au_*y*_ entities is reflected in the standard deviation. With a standard deviation of 11.6 at. %, the as-synthesized particles showed a much higher variation of Ag content than the dealloyed nanoparticles (Figure 3b), while the higher number of data points in the case of the as-synthesized particles needs to be considered. However, concerning the catalytic activity, the standard deviation and distribution width slightly increased after 0.5 cycles of dealloying compared to the as-synthesized particles (Figure 3d, f).

In correlating Ag content and activity for each individual location of the SECCM experiment (Figure S6), a trend toward higher activity at lower Ag content for the as-synthesized bimetallic particles was observed. This is expected as Au is more active toward the HER than Ag.^67^ However, the high variability of the particles is reflected in their broad populations, both for the as-synthesized particles and the particles at all four dealloying states. In simple terms, it can be said that the as-synthesized particle groups varied more in composition than in activity, while the opposite was true for the dealloyed particles, especially after the first potential cycle. This confirms that the particle composition is not the only parameter influencing the activity of nanocatalysts. After the first dealloying cycle, considering the standard deviations (Figure 3d) and the length of the box charts/whiskers (Figure 3f) for the catalytic current density, some of the statistical variation is diminished at the higher dealloying states. A decrease in average activity and a shift in its distribution after 100.5 cycles indicates a slight catalyst deactivation in line with our macroelectrode experiments (Figure 1b) and previous reports.^5^ However, the activity difference between 0.5 and 100.5 cycles is not statistically significant according to the Mann–Whitney *U* test.

In summary, we demonstrated that SECCM can be used to efficiently test the performance and statistical variability of nanocatalysts undergoing different treatments. The workflow presented here enables extensive characterization of a single batch of nanoparticles, by combining targeted modification under different conditions with catalytic testing and compositional analysis of small particle groups. By this multiscale analysis, different catalytic properties can be linked with each other; in this case screening as-synthesized and dealloyed Ag_*x*_Au_*y*_ nanoparticles with different Ag content for maximum catalytic activity toward the HER. The catalytic activity of the nanoparticles was maximized after only 0.5 cycles of dealloying and strong variations between elevated catalytic activities were found after the treatment. The Ag content was greatly decreased after 0.5 cycles and remained at ∼8 at. % upon further dealloying.

With the above analysis, nanoparticles at different dealloying states were statistically analyzed. However, this analysis does not consider the dealloying history of the particles on a particle-by-particle basis, since the different dealloying steps were still applied to separate sets of Ag_*x*_Au_*y*_ entities (within the same batch). In the next section, we aim to follow the same particles throughout the dealloying treatments, in order to identify which particles are dealloyed faster or slower than others using IL-STEM/EDX. Using this methodology, it is also possible to gain further insights into dealloying-induced morphological changes as well as the exact particle composition by elemental analysis.

### IL-STEM/EDX

The nanoparticles were characterized with IL-STEM and EDX to evaluate their size, morphology, and Ag content after four consecutive increases in dealloying cycle number. For these measurements, the Ag_60_Au_40_ particles were deposited and dealloyed on the electron beam transparent carbon electrode, made of boron-doped diamond (BDD).^51^ BDD^68^ displays outstanding electrochemical and mechanical stability for repeated IL-STEM measurements, as required herein.^48,51^

IL-STEM imaging was performed at four different states of dealloying, at 0 (before dealloying), 0.5, 10.5, and 100.5 voltammetric cycles. It was previously shown using SECCM, that after 0.5 cycles the maximum average catalytic activity had been reached (Figure 3d). For every dealloying step, the BDD electrode was contacted as the WE in a 3-electrode setup, making sure the electron beam-transparent area around the central hole was fully immersed in the electrolyte (Figure S7a).

Low-magnification STEM showed that the electron beam-transparent regions of the BDD electrode were covered with both primary (isolated) particles and particle aggregates (referred to collectively as entities, *vide supra*) after electrodeposition (Figure S8). The BDD electrode provided a stable support for the entities throughout the four dealloying steps. Repeated high-magnification IL-STEM imaging and EDX analysis were successful for a sample size of >70 Ag_*x*_Au_*y*_ entities before and after each dealloying step (Figure S9). The consecutive IL-STEM images for two regions (compared to Figure S9) are shown in Figure 4a*i* and *ii*. Morphologically, the primary particles were found to stay spherical or oval throughout the dealloying treatment. As potential cycling progresses, individual primary nanoparticles can be seen to shrink in size (example marked by yellow, dashed circle in Figure 4a*i*).

**Figure 4:**
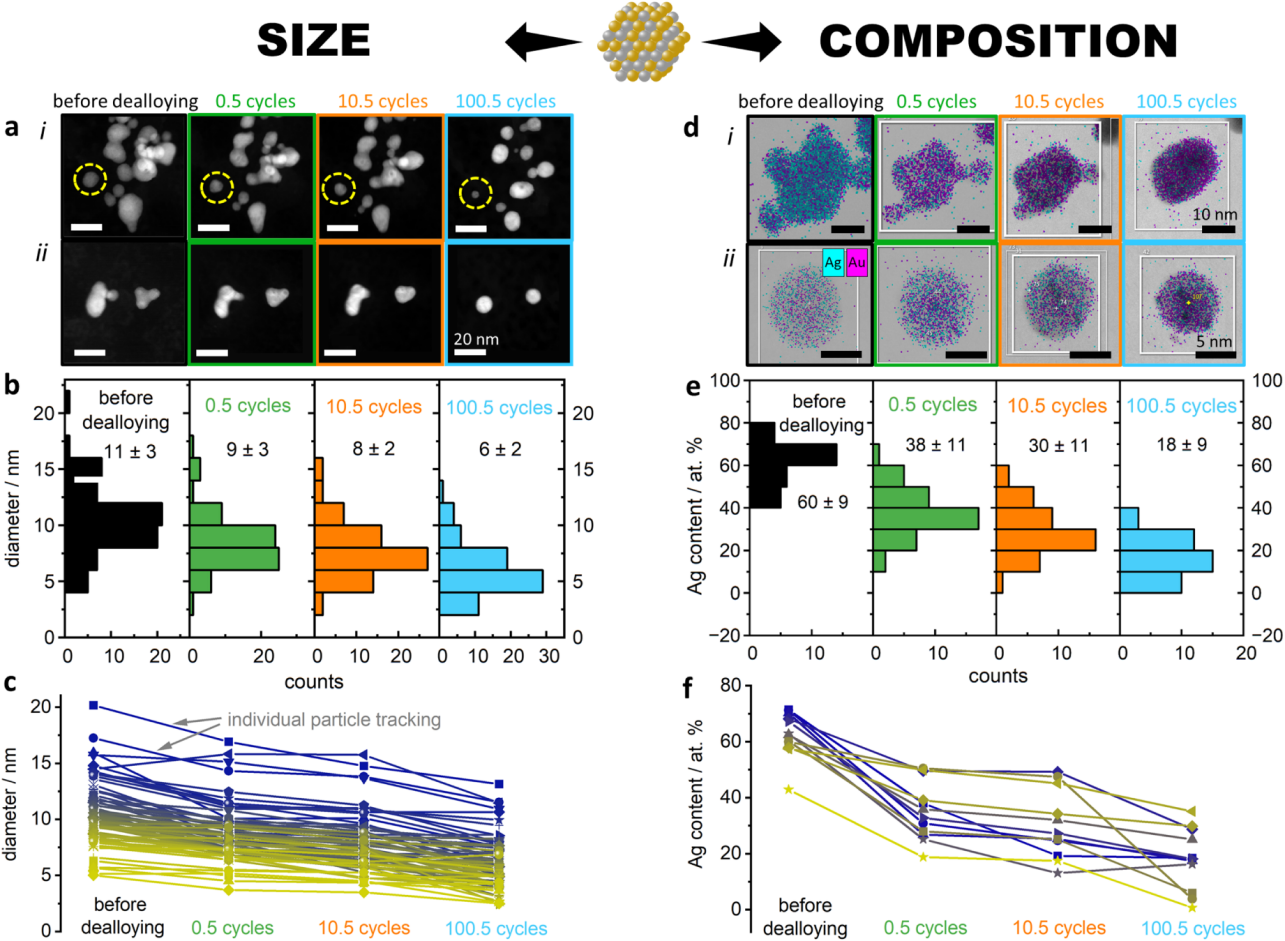
Tracking nanoparticles during dealloying. (a) Annular dark-field (ADF) STEM images of identical Ag*_x_* Au*_y_* entities at four different states of dealloying on the BDD electrode, (b) particle size distributions measured by STEM at 70 primary particles at different dealloying states in identical locations, including mean values and standard deviations, (c) primary particle diameters tracked over all dealloying states by IL-STEM (each line represents the history of an individual particle), (d) overlay of bright-field STEM and EDX mapping of a single particle aggregate (*i*) and a primary particle (*ii*) at four dealloying states, (e) statistical distributions of Ag content in dealloyed Ag*_x_* Au*_y_* entities from EDX, including mean values and standard deviation, (f) Ag content tracked over all dealloying states by IL-EDX, analogous to (c).

Using IL-imaging, the diameter of 70 primary nanoparticles was tracked over the four dealloying states (Figure 4b–c). Figure 4b represents the particle size distribution histograms. The average diameter of the as-synthesized primary Ag_60_Au_40_ particles was 11 ± 3 nm which decreased to 6 ± 2 nm (a 43 ± 13% decrease in size) after 100.5 cycles. The size distribution does not include those primary particles that disappeared during dealloying, an example of which is shown in Figure S10 (marked with blue, dashed circle). For instance, out of 25 counted primary particles, 23 remained after both 0.5 cycles and 10.5 cycles, while only 18 were found after 100.5 cycles. The missing particles are assumed to either have dissolved or detached from the electrode.

Significant particle aggregation was found in the as-synthesized particle ensemble (∼50% of all entities, see Figure S9). Upon dealloying, particles within an aggregate often merged, forming more compact structures, referred to as “coalescence” herein. Examples can be seen in Figure 4a*ii* and d*i*, as well as Figure S10. Generally, a tendency for aggregated particles to coalesce into a spherical structure was observed. The largest morphological change was found between 10.5 and 100.5 cycles. Particle coalescence is linked to a decrease in surface-area-to-volume ratio, which may account for the observed loss in mass-related catalytic activity in addition to the above-mentioned Au passivation. However, this effect will (partially) be countered by particle shrinkage as the ratio between surface area and volume should increase for decreasing particle radii.

Different to previous work on carbon-filmed Cu grids,^5^ no formation of new aggregates from primary particles was observed. The Ag_*x*_Au_*y*_ entities that did not dissolve or detach were found to be relatively stationary (Figure S9) and did not move extensively across the surface, indicating a stable interaction between the Ag_60_Au_40_ alloy and the oxygen terminated BDD electrode.^69^ Pore formation was not observed (at the magnifications used herein), in line with previous work which suggests nanoparticles must be ≥25 nm to form pores upon dealloying.^3^

The identical-location EDX mapping showed a homogeneous alloy structure throughout all Ag_*x*_Au_*y*_ entities investigated in all four dealloying states (e.g., Figure 4d, Figure S11). Dealloying experiments on nanoparticles (5 nm diameter Ag_0.77_Au_0.23_ nanoparticles) of similar size to those studied herein, using a constant dealloying potential, showed core–shell structure formation (with an Au-rich shell) as a result of superficial dealloying.^3^ This follows the theory that percolation dealloying is less likely to occur in small particles as a result of increased surface curvature.^28^ However, other works have shown that potential cycling can reinforce percolation dealloying, resulting in more homogeneous dealloying of very small nanoparticles.^7,12^ Our work confirms the latter with high statistical certainty.

As with the SECCM data, on average, the strongest compositional change to the Ag_*x*_Au_*y*_ entities was again observed after the first potential sweep. In the statistical distribution of the Ag content presented in Figure 4e, the average went from 60 ± 9 at. % Ag in the precursor particles to 38 ± 11 at. % after 0.5 cycles of dealloying. The standard deviation of the Ag content distribution was not significantly changed by dealloying, reflecting a similar variation of Ag and Au contents in the dealloyed and as-synthesized particles.

Comparing the dealloying on the BDD electrode (Figure 4e) with the local dealloying approach using SECCM (Figure 3b), the rate of total Ag loss is similar, but it is evident that using the micropipette, the Ag is leached out faster. This is likely due to the increased diffusional flux of Ag^+^ ions away from the electrode in the SECCM configuration, compared to the macroelectrode.^70^ Hence, fewer cycles are needed to yield similar degree of Ag removal, which can be seen as an advantage of SECCM, providing a more efficient route for targeted particle modification and characterization.

Using the IL-STEM/EDX results, an additional statistical variance could be unveiled by analyzing how each individual particle’s or aggregate’s size and Ag content changed between dealloying cycles. In Figure 4f, the Ag content of several individual particles (*n* = 7) and aggregates (*n* = 5) were tracked after 0.5, 10.5, and 100.5 consecutive dealloying cycles. The investigation proved that all Ag_*x*_Au_*y*_ entities underwent at least some compositional change, which also indicates good electrical contact between the electrodeposited particles and the electrode. The differences in the slopes between the “before dealloying” state and the 0.5 cycles state indicate that the Ag_*x*_Au_*y*_ entities that showed higher Ag content in the beginning (blue curves) on average lost relatively more Ag in the first dealloying step compared to others. This is expected based on previous literature,^64^ where the critical potential was found to be lower for particles with higher content of the less noble metal. Dealloying by CV would therefore result in particles with higher Ag content being exposed to potentials above their critical potentials for longer periods of time, which would lead to increased dealloying of these particles.

For the primary particles tracked by both IL-STEM and EDX throughout all four dealloying states, the relative change in size was correlated with the relative change in Ag content for primary particles (Figure 5a), from which statistical distributions of size change (Figure 5b) and Ag content change (Figure 5c) were also revealed. It is striking that there is a strong heterogeneity in the behavior of individual particles in terms of size change and Ag loss. On average, as observed for primary particles and aggregates, the highest Ag loss per cycle was found during the very first potential cycle (−40 ± 16%, Figure 5b). Further dissolution of Ag is observed with further cycling. However, the rate of loss of Ag per cycle decreased with extensive cycling, which shows that Ag dissolution is not linear over time. This behavior can be explained by the reorganization of Au surface atoms, stabilizing the remaining Ag in the bulk of the particle.^62^ Yet, some Ag loss was measured between the 10.5 and 100.5 cycles.

**Figure 5:**
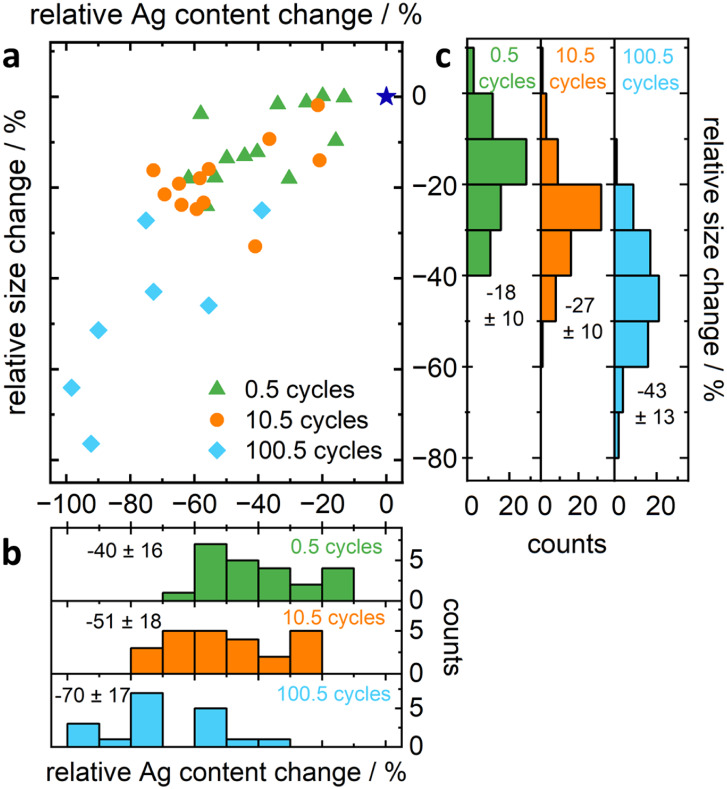
Combined IL-STEM/EDX data, (a) relative change in diameter after each dealloying step toward original size (before dealloying, represented by dark-blue star) over relative Ag content change of those primary particles that were characterized by both STEM and EDX in each state, combined with (b,c) histograms with mean values and standard deviation of relative change in (b) Ag content (including more data from primary particles and aggregates), (c) diameter (including more data from only primary particles). The underlying data is additionally available in Table S3.

The blue distribution in Figure 5c indicates a significant shrinking of the particles over the last 90 cycles. To estimate if this extensive shrinking would be expected under the assumption that only Ag and no Au was leached from the particles, estimates of the average sizes of the dealloyed particles were calculated using the average Ag content at each dealloying state from the distributions in Figure 4e. Further details are provided in S4.4, Supporting Information. It is assumed that the particles start with an average diameter of 11 nm (Figure 4b). After 100.5 cycles (average 18% Ag), the particle diameter was estimated at ∼9 nm, using the procedure in S4.4, which is larger than the mean value of the STEM measured distribution (6 nm ± 2 nm, Figure 4b). This points to dissolution of Au atoms from the alloy due to the continuous surface reorganization over 100.5 cycles. An additional explanation may be a density increase upon dealloying, which would also result in smaller particles than expected. For instance, the dealloying of carbon-supported Pt_3_Co nanoparticles resulted in a contraction of the crystal lattice.^21^ Further studies focusing on the crystal structure and possible evolution of different surface orientations—ideally in an identical-location 4D STEM approach^49^—would be highly desirable. However, the difference between the measured and theoretical diameter is too high to be explained by a density change only, and the dissolution of Au is likely to be the dominating factor.

In summary, the IL-STEM/EDX analysis has shown the strongest compositional changes occur during the first anodic sweep where the particles lose on average 40 ± 16% of their Ag. Consecutive cycling resulted in further Ag leaching with high statistical variations between Ag content and size changes of the individual particles. The spherical morphology and alloy structure of the primary nanoparticles was largely preserved over 100 dealloying cycles, as primary particles decreased in size. Coalescence of aggregated particles was observed, and the possibility of Au dissolution upon extensive cycling was highlighted.

## Conclusion

Valuable insights were gained into the statistical property–activity relationships of micelle-derived Ag_*x*_Au_*y*_ nanoparticles at different states of potentiodynamic dealloying corresponding to different activities with respect to the HER. With the Ag_60_Au_40_ nanoparticles immobilized on a GC electrode, the catalytic activity was greatly increased after 10.5 cycles in HClO_4_, while further dealloying led to a moderate deactivation of the catalyst.

In order to investigate dealloyed nanoparticles on a submicron scale, a novel two-step SECCM workflow was demonstrated, combining local dealloying with rapid screening of small groups of nanocatalysts for their mass-related catalytic HER activity. Acquiring a high number of data points, statistical activity distributions of Ag_*x*_Au_*y*_ entities in several states of dealloying were generated. While the average catalytic activity was already maximized after the first 0.5 cycles, a higher statistical variation in activity between individual groups of Ag_*x*_Au_*y*_ entities was obtained. Moreover, it was shown how SECCM can be efficiently applied as an analytical tool to determine the relative Ag content of the particles and correlate composition with activity. Compared to the experiments on the macroelectrode, fewer dealloying cycles were needed with the micropipet to yield similar percentages of Ag removal. This was likely a consequence of the increased mass transport in the SECCM configuration, highlighting that SECCM can be used as an accelerated testing platform.

Finally, using IL-STEM/EDX on a BDD electrode, the individual response to the dealloying treatment of >70 Ag_*x*_Au_*y*_ entities was analyzed. Starting with a particle size of 11 ± 3 nm, pore formation was not observed (at the magnifications used herein). Instead, the alloy structure was preserved, in contrast to the formation of a core–shell structure,^3^ indicating that percolation dealloying had occurred. The average Ag content decreased from 60 ± 9 at. % to 18 ± 9 at. % over 100.5 cycles, with the largest decrease occurring during the first 0.5 cycles. The primary particles, largely spherical in shape, decreased in size throughout all the 100.5 cycles of dealloying, resulting in sizes which suggested undesired dissolution of Au had also occurred. Particle coalescence occurred as a result of dealloying and may explain the observed deactivation of the catalysts. Tracking identical particles and aggregates throughout the four dealloying states using IL-STEM showed that Ag_*x*_Au_*y*_ entities with higher initial Ag content were found to lose relatively more Ag during the first 0.5 cycles than particles with lower initial Ag content. Over the last 90 cycles, complete dissolution or detachment of some particles from the electrode was observed.

The multiscale analysis used in this work offers a holistic approach toward an in-depth understanding of property–activity relationships of electrocatalysts, working with statistical distributions instead of single data points. Further developments and automation in this field may help to realize a tailored design of electrocatalysts based on high-throughput screening and big data.

## Experimental Section

### Reverse Micelles

Reverse micelles in toluene were synthesized as reported in previous works.^18,53,54^ For the synthesis of precursor-filled reverse block copolymer micelles, 50 mg of PS−P2VP [P1330−S2VP, Mn: PS(48500)−P2VP(70000)] (from Polymer Source) was dissolved in 5 mL of toluene as solution 1. The polymers form spherical reverse micelles in which the polar poly-2-vinylpyridine (P2VP) heads constitute the center and the nonpolar polystyrene (PS) tails extend outward. 33.23 mg of HAuCl_4_·3H_2_O and 14.35 mg of AgNO_3_ were added to 5 mL of toluene as solution 2 and then 1 mL of tetrahydrofuran was added to dissolve the Au and Ag precursors completely. The two solutions were vigorously stirred for 24 h. After that, solution 2 was added into solution 1 and stirred for 48 h.

### Sample Preparation

For all aqueous solutions, ultrapure water purified using a Barnstead GenPure xCAD Plus (Thermo Fisher Scientific, conductivity 0.055 μS cm^–1^ at 25 °C) was used. The WE, a GC plate (HTW Hochtemperatur-Werkstoffe GmbH, 15 × 15 × 0.5 mm^3^) was polished to a mirror finish in 3 steps for 3 min each with Al_2_O_3_ particle suspensions (LECO Instruments GmbH) with different particle sizes (1 μm, 0.3, and 0.05 μm). After polishing, the WE was cleaned by ultrasonication (Elmasonic S 100H) for 10 min in propan-2-ol (VWR Chemicals, ACS) and 10 min in ultrapure water, followed by drying with nitrogen. For the electrodeposition, the WE was mounted into a polytetrafluoroethylene (PTFE) cell with an O-ring of 8 mm diameter, leaving an exposed area of 0.5 cm^2^, and contacted from the bottom. Following previous work on the micellar-based electrodeposition of Ag_*x*_Au_*y*_ nanoparticles,^18^ 10 μL of the micellar solution were mixed with 320 μL of toluene (VWR Chemicals, 99.99%) and 670 μL of 50 mM tetra *n*-butylammonium hexafluorophosphate (TBAPF_6_, Alfa Aesar, 98%) in 1,2-dichloroethane (DCE, Carl Roth, ≥99%). The toluene was filtered with a 200 nm pore size PTFE filter prior to use. After filling the solution into the PTFE cell, a platinum wire was inserted as the quasi-reference electrode (QRE) and a graphite rod (*d* = 6 mm) as the counter (CE). Using a potentiostat (PalmSens4, PalmSens BV), a constant voltage of −1.3 V vs Pt was applied to the WE for 30 min. This procedure had previously been found suitable to form alloy-type Ag_*x*_Au_*y*_ nanoparticles.^18^ After the deposition was completed, the WE was washed three times in toluene, three times in acetone (Sigma-Aldrich, ACS, >99.5%), two times in isopropanol, and four times in ultrapure water to remove the residues from the micellar polymer, before drying under a nitrogen stream.

### Voltammetry on a Macroelectrode

Before investigating the microscopic features of dealloyed Ag_*x*_Au_*y*_ particles, macroscopic electrochemical measurements were performed in a conventional beaker experiment. These measurements were conducted in a Faraday cage using a potentiostat (Autolab PGSTAT 128N, Metrohm). After preparation as described above, the nanoparticle-functionalized WE was mounted into a PTFE cell, identical to the one used for the electrodeposition. A graphite rod (*d* = 6 mm) was used as a CE and either an in-house built silver–silver chloride reference electrode (Ag|AgCl|3 M KCl, 0.21 V vs standard hydrogen electrode, SHE) or a mercury–mercurous sulfate reference electrode in saturated K_2_SO_4_ (MSE sat., 0.65 V vs SHE, SI Analytics GmbH) for chloride-free electrolytes. For electrochemical dealloying, the silver was electrochemically dissolved in 1.0 M HClO_4_ (pH = 0.3, diluted from 70%, VWR Chemicals, ACS) by cyclic voltammetry using either 0.5, 10.5, or 500.5 cycles. The potential was swept from 0.0 to 1.25 V vs Ag|AgCl at a scan rate of 1.0 V s^–1^. Every dealloying step ended with half a cycle at the anodic upper potential (=one linear sweep) to avoid silver redeposition during the back scan. The electrocatalytic activity of the particles toward hydrogen evolution was measured in 0.5 M H_2_SO_4_ (pH = 0.5, diluted from 95%, Fisher Scientific, analytical reagent grade) after degassing the solution with Ar for 15 min. Using linear sweep voltammetry between −0.6 V and −1.4 V vs MSE reference electrode with a scan rate of 2 mV s^–1^, the polarization curves were averaged over three measurements, respectively. The same solution was used to estimate the electrochemically accessible surface area (ECSA) by capacitive cycling at six different scan rates (Figure S3). Finally, Ag_*x*_Au_*y*_ nanoparticles were electrochemically dissolved by anodic stripping in HCl (Sigma-Aldrich, 37%, reagent grade, diluted to 0.1 M concentration (pH = 1.1)), by CV between −0.2 and 1.2 V vs Ag|AgCl at 25 mV s^–1^ scan rate to estimate the mass of the particles. Due to this step, it was necessary to use individual nanoparticle-functionalized WEs for each dealloying step, corresponding to different numbers of dealloying cycles.

### SECCM

The working principle and setup of typical SECCM experiments have been described previously.^33,43^ Further information about the instrumentation of the home-built workstation can be found in the Supporting Information S3.1.

A schematic of the combined SECCM experiment is depicted in Figure 2. At first, a single-barrel micropipet with a ∼65 μm diameter tip was pulled from a borosilicate capillary (Sutter Instrument, BF100–50–10) using a PC-100 heated-coil puller (Narishige) and backfilled with 1.0 M HClO_4_ (pH = 0.6, Sigma-Aldrich, 70%, 99.999% trace metal basis). A leak-free Ag|AgCl|sat. KCl electrode (Innovative Instruments, Inc.) with a 0.5 mm diameter was inserted into the back of the pipet and contacted as the quasi-reference counter electrode (QRCE). The nanoparticle-functionalized GC-plate was prepared as described above (“Sample Preparation”) but here using 10 min deposition time, instead of 30 min, to achieve a wider dispersion of nanoparticles. It was contacted as the WE.

The probe was gradually approached to the WE surface at a rate of 1 μm s^–1^ using a piezo actuator, while a potential of 0.0 V was applied between the QRCE and the WE, and the current at the WE was continuously monitored using a custom-built current amplifier. The approach was stopped once a preset current threshold was reached, which indicated the contact between the droplet at the probe’s tip and the WE’s surface, closing the electric circuit. Then, the probe was held stationary while the surface potential was cycled between 0.0 and 1.25 V for electrochemical dealloying, using a scan rate of 1.0 V s^–1^, analogous to the dealloying treatment on a macroelectrode. After the desired cycle number was reached, the probe was retracted applying 0.7 V vs Ag|AgCl, to avoid any Ag redeposition or additional dealloying during the retraction. To create four areas with nanoparticles at different dealloying states, the pipet was approached at four positions in a square pattern with 200 μm distance between the points. In each point of the square, a different number of cycles was applied (0.5, 1.5, 10.5, and 100.5 cycles, respectively), while the particles outside the wetted areas remained untreated. After each of the four approaches with the pipet, the sample was gently rinsed with ultrapure water and dried under nitrogen flow to prevent any crystallization of the highly concentrated electrolyte on the surface.

To characterize the nanoparticles at the five different states of dealloying (four contact areas from scan with the large pipet, plus the surrounding untreated particles), a second SECCM experiment was performed in the same area on the sample. For that, the electrode was placed in a suitable environmental chamber, with a hole for the pipet, filled with Ar. A smaller pipet with a tip diameter of ∼400 nm was pulled using a CO_2_ laser puller (P-2000, Sutter Instrument) from a borosilicate capillary (Havard Apparatus, GC120F-10). Table S1 shows the respective laser puller parameters.

The smaller pipet was filled with 50 mM HCl solution (pH = 1.7, Sigma-Aldrich, 37%, reagent grade) and a leak-free Ag|AgCl electrode was inserted. Then the probe was approached to the surface while applying a −0.5 V bias. As soon as the droplet contacted the WE, 3 CV cycles were recorded, between −0.5 V and −1 V at 0.5 V s^–1^ scan rate, to measure the activity toward HER; followed by 1 CV cycle, with 1.2 V upper anodic and −0.6 V lower cathodic potential, to dissolve and redeposit the material (see Figure 2c,d). This procedure was repeated at 625 individual points on the surface, covering a square area of ∼500 μm side length, including all four dealloyed spots and the surrounding untreated areas. Investigating the measured area by post-SECCM scanning electron microscopy (SEM) did not reveal the size of the small footprints, but a control experiment under the same conditions resulted in a footprint diameter of ∼650 nm (see Figure S4). Considering the nanoparticle coverage, we can assume that on average about 5–10 particles were contacted in each landing point. From the CV data, the local catalytic activity and Ag content were evaluated as described in Section S3.1 using MATLAB routines.

### IL-STEM/EDX

For the structural characterization of the dealloyed and as-synthesized Ag_*x*_Au_*y*_ particles, an in-house prepared electron-beam transparent BDD electrode was used, the fabrication of which, along with the material used, is described in full in ref 51.

The micelle-assisted nanoparticle electrodeposition was performed in a glass vessel, using the same QRE and CE as mentioned above. The BDD electrode (WE) was contacted with a metal clamp before placing it in the organic solution with micromanipulators (analogous to Figure S7a) until the central hole of the electrode was fully immersed, and the contact was kept out of solution. Carbon ink (838AR MG Chemicals, UK) was applied to the laser patterned area of the electrode to enhance electrical contact. The chronoamperometry was conducted as mentioned above (see “Voltammetry on a Macroelectrode”), using a potentiostat (CompactStat.h, IVIUM) and 5 mL organic solution, comprising 3.335 mL of 50 mM TBAPF_6_ (Sigma-Aldrich, 98%) in DCE (Sigma-Aldrich, ≥99%), 1.65 mL of filtered toluene (Fisher Scientific, laboratory reagent grade) and 15 μL of micellar solution. After the deposition, the BDD electrode was washed and dried in the same way as the GC electrode. To gradually dealloy the micelle-nucleated particles on the electrode, it was contacted with carbon ink and placed in a three-electrode setup together with a leakless Ag|AgCl|sat. KCl RE and a Pt mesh serving as CE (see Figure S7a). Using a potentiostat, the particles were dealloyed with 0.5, 10.5, and 100.5 cycles in 1.0 M HClO_4_ (pH = 0.6, diluted from 70%, Sigma-Aldrich, 99.999% trace metal basis) on the same BDD electrode (see CVs in Figure S7b) and imaged by STEM/EDX after each step of the treatment (including initial imaging before any dealloying).

Identical-location STEM was performed using a double aberration ion-corrected ARM200F atomic resolution STEM operating at 200 kV. EDX spectra were acquired using a 100 mm^2^ windowless EDX detector (Oxford Instruments, UK). The EDX spectra were analyzed using AZtec software (Oxford Instruments, UK) to extract the atomic ratio of Ag and Au in the nanoparticles from the *L_α_* 1 lines, respectively.

## Supplementary Material



## Notes

Further experimental details, including precharacterization of reverse micelles for nanoparticle synthesis, normalization methods, and details on SECCM instrumentation and data analysis, post-SECCM SEM/EDX analysis, alternative representation of SECCM data, details on IL-STEM/EDX analysis and supporting IL-STEM/EDX data, calculation of theoretical nanoparticle size from EDX composition, (current density vs Ag content) (PDF)

## Abbreviations

ADF: annular dark field
AFM: atom force microscopy
BDD: boron-doped diamond
CE: counter electrode
CV: cyclic voltammetry
ECSA: electrochemically accessible surface area
EDX: energy-dispersive X-ray spectroscopy
GC: glassy carbon
HER: hydrogen evolution reaction
IL-STEM: identical-location scanning transmission electron microscopy
MSE: mercury–mercurous sulfate electrode
PS−P2VP: polystyrene-*b*-poly­(2-vinylpyridine)
PTFE: polytetrafluoroethylene
QRCE: quasi-reference counter electrode
QRE: quasi-reference electrode
RE: reference electrode
SECCM: scanning electrochemical cell microscopy
SEM: scanning electron microscopy
WE: working electrode
