# Supplementary material for: Multiscale Analysis of Electrochemical Dealloying of Bimetallic Nanoparticles to Tune Catalytic Activity
Source: ACS Appl Mater Interfaces. 2026 Apr 13;18(15):21964–77. doi: 10.1021/acsami.6c00288 (PMC13107377; doi:10.1021/acsami.6c00288)
Supplement: Supplementary file 1 [file am6c00288_si_001.pdf]

Supporting Information

Multiscale Analysis of Electrochemical Dealloying of Bimetallic Nanoparticles to Tune Catalytic Activity

Johanna Angona[1,2], Dimitrios Valavanis[2], Daniel Houghton[2,3], Fengli Yang[4], Thaís Schroeder Rossi[1], Jan Clausmeyer[1], Beatriz Roldán Cuenya[4], Julie V. Macpherson[2,3,*], Patrick R. Unwin[2,3,*], Kristina Tschulik[1,5,*]

[1]Analytical Chemistry, Ruhr University Bochum, 44801 Bochum, Germany
[2]Department of Chemistry, University of Warwick, Coventry CV4 7AL, United Kingdom
[3]Hartnoll Centre for Experimental Fuel Technologies, University of Warwick, Coventry CV4 7AL, United Kingdom
[4]Department of Interface Science, Fritz-Haber Institute of the Max-Planck Society, 14195 Berlin, Germany
[5]Max-Planck-Institut für Nachhaltige Materialien GmbH, 40237 Düsseldorf, Germany

* To whom correspondence should be addressed. Email: Kristina.Tschulik@rub.de, P.R.Unwin@warwick.ac.uk, J.MacPherson@warwick.ac.uk

Contents

S1. Pre-Characterization of PS–P2VP Reverse Micelles and Generated Ag_xAu_y Nanoparticles ... 3

S2. Evaluation of Current-Potential Curves ... 5

S2.1. Determination of Ag Content and Total Mass (Ag+Au) from Anodic Stripping ... 5

S2.2. Normalization of the Catalytic Current ... 5

S3. Details on SECCM Measurements ... 7

S3.1. SECCM Instrumentation and Data Analysis ... 7

S3.2. Post-SECCM SEM and EDX Analysis ... 8

S3.3. Correlation of Activity and Ag Content from SECCM ... 9

S4.1. Dealloying of Ag_xAu_y Nanoparticles on BDD Electrode ... 15

S4.2. Coverage of As-Deposited Particles on BDD Electrode for IL-STEM/EDX ... 16

S4.3. Morphological Changes to Ag_xAu_y Entities during Dealloying ... 17

S4.4. Calculation of Theoretical Particle Size from EDX Composition ... 18

S4.5. Example for EDX Analysis ... 19

S4.6. Changes in Ag Content and Diameter over Dealloying ... 20

References ... 22

S1. Pre-Characterization of PS–P2VP Reverse Micelles and Generated Ag_xAu_y Nanoparticles

For atomic force microscopy (AFM) measurements, the solution of $HAuCl_4$ and $AgNO_3$-filled PS–P2VP reverse micelles solvated in toluene was further diluted in toluene by 1:7 and drop-casted onto a Si wafer. The Si wafer had been cleaned prior to the experiment by dipping into isopropanol. While the solvent evaporated, the reverse micelles stayed intact and in contact with the surface. AFM mapping presented in Figure S1a was performed under ambient air on a Bruker BioScope Resolve AFM, using PeakForce Quantitative Nanomechanical Mapping (PF-QNM) mode, operating at 2 kHz resonant frequency, where the scan rate was 0.5 Hz, the peak force setpoint was 1.4 nN, and the peak force amplitude was 140 nm. A ScanAsyst-Air probe from Bruker with triangular geometry (nominal radius = 2 nm), a spring constant of 0.4 N m^{-1}, and a resonance frequency of 70 kHz was employed. Figure S1b shows the size distribution obtained from the AFM data of 77 reverse micelles. This size distribution was obtained by measuring the width from line scans across individual reverse micelles. The average width strongly deviated from the average height (18 $\pm$ 3 nm), indicating that the shape of the reverse micelles sitting on a substrate is more quasi-hemispherical than spherical. Therefore, the width was chosen as a measure for the reverse micelle diameter but is may still not accurately reflect the size of the reverse micelles when free in solution.

Additional STEM imaging of the precursor-filled reverse micelles was performed using a JEOL JEM-2800 electron microscope operating at 200 kV. For that, the reverse micelle solution was drop-casted onto an Ultrathin C film on Lacey carbon support film, 400 mesh Cu grid (Ted Pella). As can be seen in Figure S1c, the reverse micelles were found to be spherical. The small bright spots that could be found in every reverse micelle are likely caused by electron-beam induced nucleation of the precursor salts. Figure S1d shows the size distribution generated by STEM imaging of 870 reverse micelles and using the Software ImageJ. The smaller average diameter compared to the size distribution generated by AFM imaging might stem from further drying of the reverse micelles in the vacuum of the microscope chamber.

Figure S2a is an exemplary STEM image of the Ag_xAu_y nanoparticles, generated from the reverse micelles shown in Figure S1, by electrosynthesis at a BDD electrode. Characterization by STEM was performed as described in the experimental section, and the size distribution in Figure S2b was obtained from 70 primary particles using the software ImageJ. A monomodal size distribution with an average nanoparticle diameter of 11 nm $\pm$ 3 nm was found – hence, the nanoparticles are in the range of 20 – 40 % the size of the original reverse micelles.

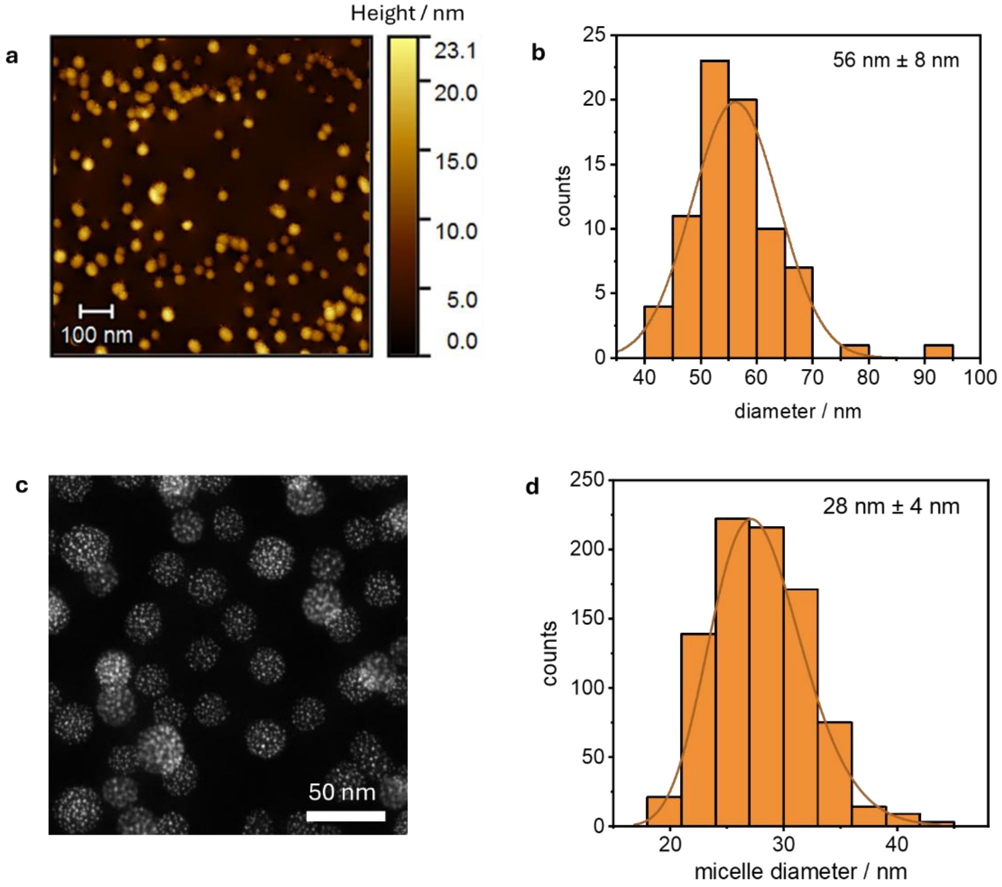

Figure S1. $HAuCl_4$ and $AgNO_3$-filled PS–P2VP reverse micelles before electrosynthesis. (a) AFM image of drop-casted reverse micelles on a GC plate, (b) size distribution from 77 reverse micelles measured by AFM, measured by the width (x, y) of the quasi-hemispherical objects, (c) STEM image of spherical reverse micelles, (d) size distribution generated from 870 reverse micelles.

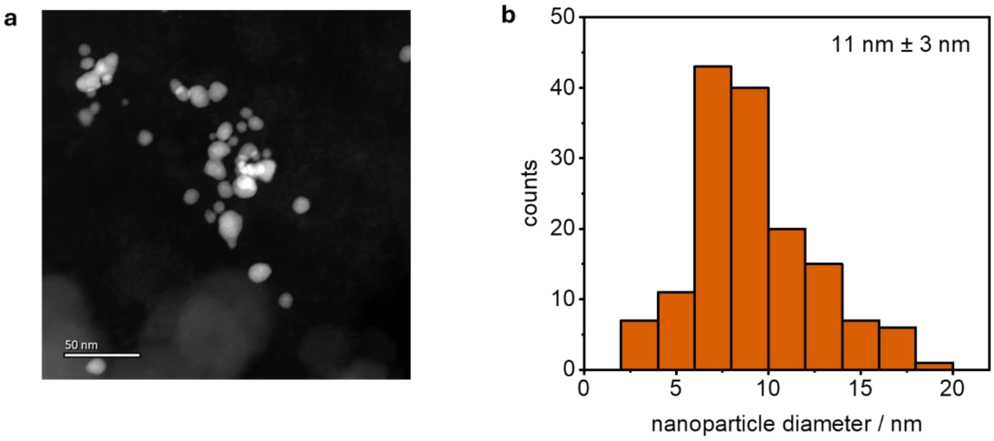

Figure S2. Ag_xAu_y nanoparticles after electrosynthesis from reverse micelles. (a) Typical STEM image of (partly aggregated) Ag_xAu_y nanoparticles, (b) size distribution generated from 70 primary nanoparticles.

S2. Evaluation of Current-Potential Curves

S2.1. Determination of Ag Content and Total Mass (Ag+Au) from Anodic Stripping

Anodic stripping of Ag and Au in HCl was performed both on the nanoparticle-functionalized macroelectrode (Figure 1c) and on small groups of Ag_xAu_y entities using SECCM (Figure 2d). By integrating the oxidation peaks A4 and A5 (for Ag) as well as A6 (for Au) and dividing by the scan rate υ, the transferred charges Q_{Ag} and Q_{Au} related to Ag and Au dissolution during anodic stripping in HCl were determined in each location. Further calculations were made based on Faraday's law[1,2] as follows

$$n_{\mathrm{Ag}} = \frac{Q_{\mathrm{Ag}}}{F \cdot z} = \frac{Q_{\mathrm{Ag}}}{F \cdot 1} \quad (1)$$

$$n_{\mathrm{Au}} = \frac{Q_{\mathrm{Au}}}{F \cdot 1.9} \quad (2),$$

where F is the Faraday constant, n_{Ag} and n_{Au} are the mole numbers of Ag and Au, respectively. The number of electrons transferred per metal atom z was assumed to be 1 for Ag[3] and 1.9 for Au.[4,5] F is the Faraday constant with $F \approx 96485\ \mathrm{C\ mol^{-1}}$.[6] The Ag content in the alloy, p_{Ag}, was thus calculated by

$$p_{\mathrm{Ag}} = \frac{n_{\mathrm{Ag}}}{n_{\mathrm{Ag}} + n_{\mathrm{Au}}} \quad (3),$$

and the mass of the dissolved nanoparticles $m_{\mathrm{Ag+Au}}$ for current normalization was calculated by

$$m_{\mathrm{Ag+Au}} = n_{\mathrm{Au}} M_{\mathrm{Au}} + n_{\mathrm{Ag}} M_{\mathrm{Ag}} \quad (4)$$

with the molar masses of Au[7] $M_{\mathrm{Au}} \approx 196.97\ \mathrm{g\ mol^{-1}}$ and Ag[8] $M_{\mathrm{Ag}} \approx 107.87\ \mathrm{g\ mol^{-1}}$.

S2.2. Normalization of the Catalytic Current

For the evaluation of macroscopic activity and SECCM-probed activity data, a suitable normalization had to be chosen for both methods. We chose to normalize our data by the mass of the particles present in the respectively probed area, derived by integration of the anodic stripping peaks in HCl, following S2.1. For experiments performed on a nanoparticle-functionalized macroelectrode, we additionally derived the ECSA by the capacitive cycling method. For that, the nanoparticle-functionalized working electrode was cycled in 0.5 M degassed H_2SO_4 (pH = 0.5, Fischer Scientific, 95 %, analytical reagent grade) in a small potential window (from − 0.15 V to − 0.25 V vs. MSE (sat. K_2SO_4)) at six different scan rates (5, 10, 25, 50, 100, and 200 mV/s). The capacitive current ΔI_{c} was measured at − 0.2 V vs. MSE (sat. K_2SO_4) and plotted against the scan rate ν to derive the capacitance c from the slope of the linear fit. The result was compensated by the capacitance of the blank GC plate to yield the capacitance of the particles alone. Then we assumed the specific capacitance c_{spec} to be 17 µF/cm^2 – close to that of nanoporous gold[9] – to estimate the ECSA from ECSA = $\frac{c}{c_{\mathrm{spec}}}$.[10] Using this method for current normalization is regarded to be less accurate for multi-component systems as the individual compounds

can have differing specific capacitances. However, Figure S3 comprising the linear sweep voltammograms for the HER activity at different dealloying states shows that both normalizations – by mass and by ECSA – yield the same qualitative result, specifically that the catalytic activity is maximized after 10.5 cycles. The only difference is that after longer treatments the mass-related activity is higher than the surface-related activity, which might point to surface roughening under catalytic conditions, which would lead to a higher ECSA. Using the mass-related activity for further evaluation, we include any roughening effects into the intrinsic activity of the material. However, the effect of increased catalytic activity after dealloying was so prominent that it could even be observed before any data normalization.

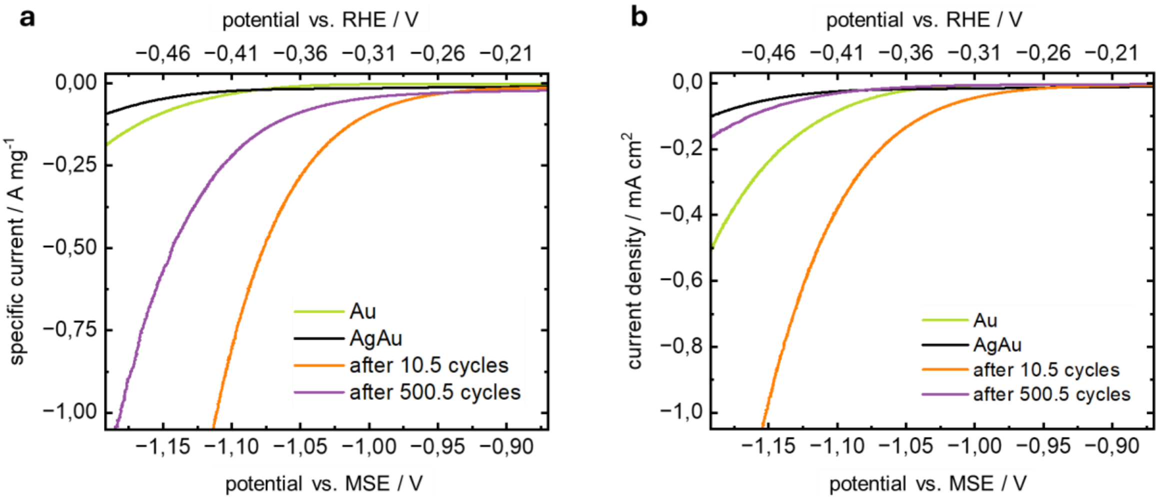

Figure S3. Linear sweep voltammetry in 0.5 M H_2SO_4 at 2 mV/s scan rate of reverse micelle-nucleated $Ag_{60}Au_{40}$ particles at three states of dealloying, compared to reverse micelle-nucleated Au nanoparticles, current normalized by (a) total mass of particles (from anodic stripping), (b) ECSA (from capacitive cycling).

S3. Details on SECCM Measurements

S3.1. SECCM Instrumentation and Data Analysis

SECCM experiments were conducted at a home-built workstation in a single-barrel configuration (two-electrode set-up, with the quasi-reference counter electrode (QRCE) inside the filled pipette probe, and the substrate connected as the working electrode (WE). The workstation comprised a custom-built current amplifier, a linear piezoelectric actuator (P-753.3CD, Physik Instrumente, Karlsruhe, Germany) for vertical movement (*z*-axis) of the pipette and a two-axis (*x*–*y*) piezoelectric actuator (P-625.2CD, Physik Instrumente) for lateral movement of the sample. The devices were controlled by a PC with an FPGA card (PCIe 7852R, National Instruments, Austin, USA) using the publicly available Warwick Electrochemical Scanning Probe Microscopy (WEC-SPM) platform[11] on a LabVIEW 2019 interface (National Instruments). Nanopipettes were pulled using a Sutter P-2000 CO_2 laser puller. The parameters for pulling pipettes with ~ 400 nm diameter tips from borosilicate capillaries (Havard Apparatus, GC120F-10) are given in Table S1.

Table S1. Pulling parameters for nanopipette fabrication (~ 400 nm diameter tip) with CO_2 laser puller Sutter P-2000.

	HEAT	FIL	VEL	DEL	PUL
Line 1	330	3	30	220	-
Line 2	300	3	40	180	120

Before starting an experiment, the pipette tip was placed at a ~50 μm distance from the area of interest using micro-positioners (M-461, Newport, USA), and monitored by a digital camera (PL-B776U, PixeLink, Ottawa, Canada) and a cold light source (MI-150, Edmund Optics, Mainz, Germany). Then the automated approach was commenced, using the piezoelectric actuator controlled by the WEC-SPM software. For experiments under argon environment, the sample and pipette tip were positioned inside a home-built compartment to which a continuous flow of humidified argon was applied, as described previously.[12] The set-up was placed inside a Faraday cage that was fixed on a vibration-dampening optical table (RS2000 and S-2000A-423.5 automatic levelling isolators, Newport, USA).

Evaluation of the SECCM data, including integration of the dissolution peaks for calculation of the Ag content p_{Ag} and mass of the dissolved nanoparticles $m_{\mathrm{Ag+Au}}$ (following the method described in section S2.1) at each location in the SECCM measurement, was done by means of a MATLAB script (MATLAB version 2023b). Examples of the CV curves from the SECCM experiment are shown in Figure 2d.

As can be seen in the stripping CV in Figure 2d, the two dissolution peaks A5 and A6 usually showed some overlap with each other and with the following current plateau indicative of AuO formation. This made the (automated) integration difficult, and it is expected that a fraction of the underlying charge might have been neglected, causing a systematic error to the absolute values of the measured mass.

Hence, the mass-normalized specific current for the catalytic activity towards HER presented in Figure 3c and d can be used without concerns for relative comparison of the catalytic activities, but the absolute values should not be used for comparison with the literature.

S3.2. Post-SECCM SEM and EDX Analysis

The SECCM-probed area on the nanoparticle-functionalized electrode was additionally imaged by scanning electron microscopy (SEM). Furthermore, point-EDX measurements were performed on multiple Ag_xAu_y entities (isolated particles and aggregates) in each of the four dealloyed areas and the non-treated area in the middle of the square. SEM and EDX were carried out with a Jeol JSM-7200F microscope, operating at 10 kV acceleration voltage and 14 µA probe current, using the lower electron detector (LED) for SEM and the Oxford AZtecEnergy X-MaxN 80 mm^2 – SDD detector for EDX.

The four darker spots in Figure S4a were identified as the large footprints from the first SECCM experiment. Due to the high coverage of entities (see Figure S4b) and electrochemical stability of the GC substrate, the small footprints from the second SECCM experiment could not be found. However, the experiment was repeated on a GC electrode with a lower entity loading (by diluting the reverse micelle solution 10 times), keeping all other conditions identical (pipette parameters, electrolyte, potential range). This control experiment yielded a footprint diameter of 650 nm (Figure S4c). Considering the original coverage, we can assume that multiple (~ 5–10) Ag_xAu_y entities were confined in each electrochemical cell in the SECCM experiment with the 400 nm diameter pipette.

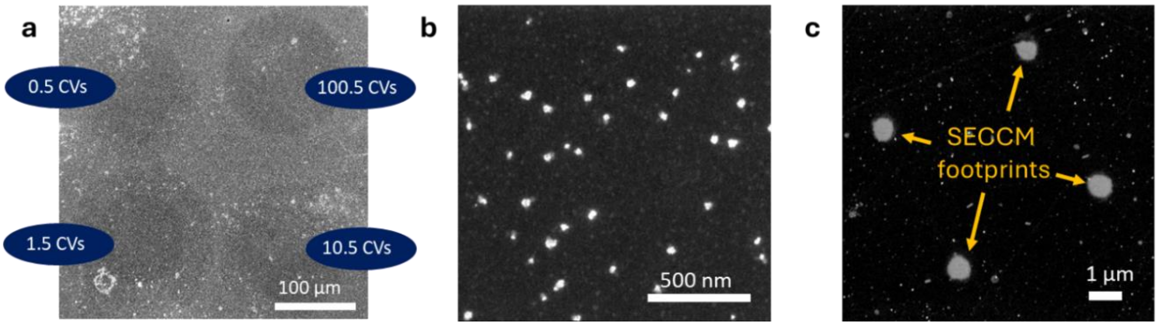

Figure S4. SEM images of the SECCM-probed area on the nanoparticle-functionalized electrode at (a) low magnification showing four big footprints from the first SECCM experiment for local dealloying, (b) higher magnification showing the coverage of Ag_xAu_y entities (c) footprints from SECCM experiment conducted under conditions identical to the second SECCM experiment (400 nm diameter tip), but with lower nanoparticle loading.

Point-EDX measurements inside the dealloyed and non-treated areas were performed to confirm the statistical distributions of the Ag content in each dealloying state, as achieved by local dealloying treatments with the micropipette (Figure S5). For each dealloying step, ~ 20 Ag_xAu_y entities were

randomly chosen inside the respective dealloyed (wetted) area as shown in Figure S5a. Figure S5b represents an exemplary EDX spectrum with the assigned fitted peaks. As can be seen in the spectrum, the EDX signal from single particles in the SEM is very weak. Therefore, STEM-EDX measurements were done in the next section to obtain the particle composition with much higher precision.

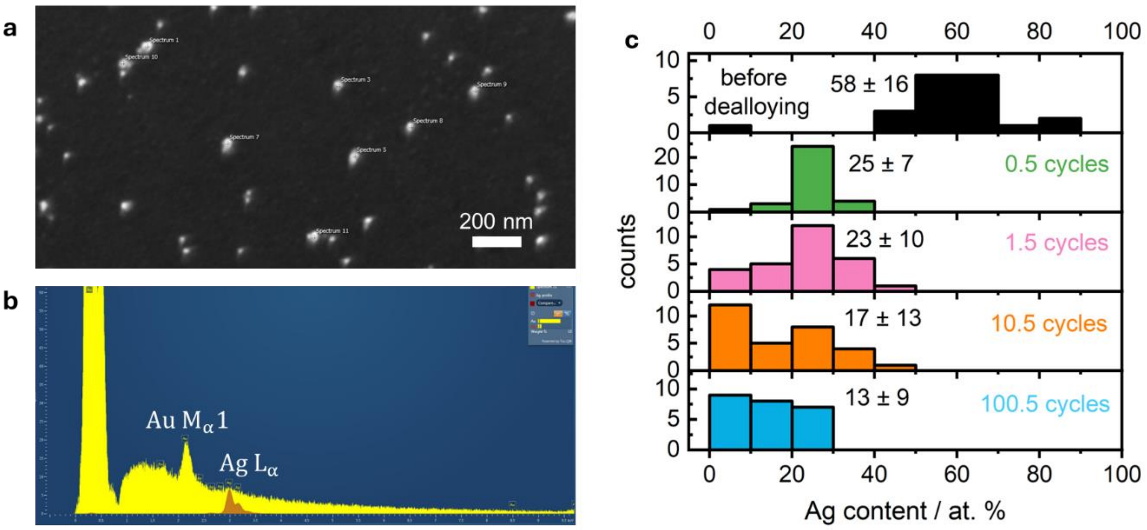

Figure S5. Post-SECCM point-EDX measurements at Ag_xAu_y entities inside the untreated and locally dealloyed areas on the nanoparticle-functionalized electrode; (a) exemplary SEM image with randomly chosen positions for point-EDX measurements, (b) exemplary point-EDX spectrum in the 0.5 cycles dealloyed area, zoomed in on the $Au\ M_\alpha 1$ and $Ag\ L_\alpha$ peaks, (c) Ag content distributions for confirmation of SECCM data, obtained by point-EDX measurements.

The post-SECCM EDX measurements were summarized in composition histograms presented in Figure S5c.Compared to the histograms in Figure 3b, obtained by integration of the anodic stripping peaks, the point-EDX method gave higher average Ag contents of the dealloyed particles, but a similar value for the non-treated particles (58 % $\pm$ 16 % for electrochemically measured Ag content vs. 61 % $\pm$ 12 % for Ag content measured by EDX). This may be related to the low lateral resolution of the SEM-EDX method that might include signals from the non-treated areas around the spots of dealloyed particles. It may also point to an underestimation of the Ag content in the SECCM experiment, possibly caused by an additional charge contribution of stabilized Ag that did not appear as a clear peak in the CV but was hidden underneath the Au peak or appeared as a background charge.

S3.3. Correlation of Activity and Ag Content from SECCM

The SECCM data from the experiment with the 400 nm diameter tip yielded the Ag content and mass-related activity towards the HER of the dealloyed and untreated nanoparticles. Table S2 contains

this data. For further evaluation, both parameters were correlated for each landing point ("pixel" in the SECCM-probed array) individually, each containing one batch of few Ag_xAu_y entities (Figure S6).

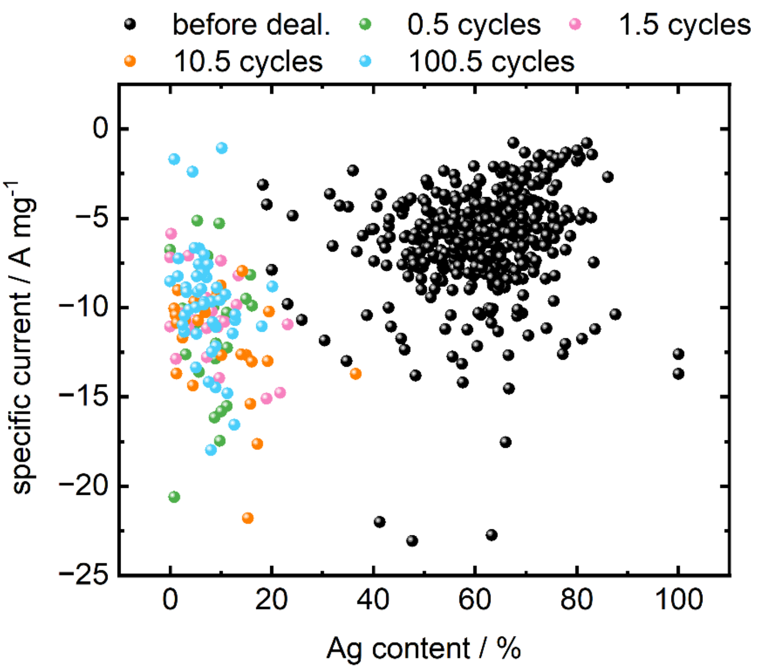

Figure S6. Correlation of SECCM data for catalytic activity and Ag content from Figure 3. Every data point is related to one landing point in the SECCM experiment with the 400 nm diameter tip, in which both activity and Ag content of small groups of Ag_xAu_y entities were measured electrochemically.

Table S2. Combined data from SECCM experiment with ~400 nm pipette tip (data presented in Figure 3), including estimated Ag content (at.%, with an estimated error of ~ 0.3 at.%) and mass-related current density j_{mass} (A mg^{-1}).

**as-synthesized**		**0.5 cycles**		**1.5 cycles**		**10.5 cycles**		**100.5 cycles**	
**Ag at.%**	**j_{mass} / A mg^{-1}**	**Ag at.%**	**j_{mass} / A mg^{-1}**	**Ag at.%**	**j_{mass} / A mg^{-1}**	**Ag at.%**	**j_{mass} / A mg^{-1}**	**Ag at.%**	**j_{mass} / A mg^{-1}**
60	-3.64	11	-15.51	9	-11.09	15	-12.65	8	-17.98
65	-7.32	6	-13.59	13	-9.86	3	-10.67	9	-14.48
66	-3.26	9	-9.97	10	-7.38	6	-8.15	18	-11.05
58	-5.96	1	-20.60	5	-10.70	1	-10.05	13	-10.71
63	-7.17	10	-17.47	19	-15.09	4	-14.37	3	-10.45
70	-1.32	7	-7.11	13	-8.20	2	-11.69	20	-8.82
69	-4.31	2	-10.05	22	-14.77	16	-15.39	9	-8.88
74	-6.24	3	-12.61	10	-10.75	2	-9.03	8	-9.66
74	-5.56	16	-9.89	0	-11.06	1	-10.42	7	-9.70
74	-11.16	10	-5.28	1	-12.88	1	-10.87	7	-7.91
76	-8.21	9	-16.15	4	-11.23	17	-17.63	4	-10.11
74	-5.58	5	-10.85	2	-10.30	19	-12.99	7	-8.31
69	-8.38	0	-6.76	10	-13.94	16	-13.02	6	-6.69
66	-8.55	9	-12.02	8	-10.21	37	-13.71	2	-8.25
59	-8.77	5	-5.13	11	-10.80	6	-10.72	12	-11.45
53	-6.79	9	-12.85	0	-7.18	10	-9.54	6	-7.57
61	-7.35	10	-15.82	4	-7.09	7	-10.28	3	-11.36
20	-7.88	11	-12.25	7	-11.16	15	-21.78	1	-1.70
64	-5.97	15	-9.51	0	-5.86	14	-12.62	5	-11.47
55	-7.60	16	-8.16	23	-10.93	19	-10.22	4	-2.40
60	-12.16	11	-10.28	7	-9.43	9	-9.20	10	-1.06
35	-4.36			7	-12.78	14	-7.95	3	-8.87
58	-6.89					10	-12.67	9	-11.07
69	-7.62					9	-9.48	9	-12.16
72	-7.64					7	-10.05	6	-8.09
69	-4.84					5	-9.67	5	-9.12
46	-12.35					1	-13.70	0	-8.53
56	-7.31					10	-8.75	11	-14.80
50	-3.96							11	-9.28
61	-4.69							5	-9.98
70	-11.56							5	-8.24
81	-5.07							7	-7.58
68	-3.67							8	-12.48
70	-5.52							8	-10.81
60	-8.23							13	-16.56
67	-7.77							2	-10.97
49	-7.47							5	-13.35
65	-5.65							6	-8.95
63	-22.73							3	-9.13

as-synthesized		0.5 cycles		1.5 cycles		10.5 cycles		100.5 cycles	
Ag at.%	j_{mass} / A mg^{-1}	Ag at.%	j_{mass} / A mg^{-1}	Ag at.%	j_{mass} / A mg^{-1}	Ag at.%	j_{mass} / A mg^{-1}	Ag at.%	j_{mass} / A mg^{-1}
59	-3.47							7	-9.84
59	-7.15							2	-7.25
63	-8.76							5	-6.65
53	-8.42							8	-14.16
54	-6.93							13	-10.40
58	-6.51							7	-7.03
49	-7.72							10	-9.58
68	-6.61								
67	-2.72								
47	-6.91								
69	-5.66								
63	-6.36								
78	-12.03								
59	-7.00								
68	-5.60								
75	-6.94								
80	-1.78								
84	-11.21								
64	-9.00								
61	-5.09								
61	-6.68								
56	-7.81								
70	-2.65								
59	-8.16								
49	-7.80								
64	-11.32								
56	-6.96								
68	-3.67								
72	-3.09								
53	-8.37								
57	-4.81								
61	-4.64								
68	-3.28								
54	-5.15								
68	-5.49								
72	-4.90								
68	-6.59								
70	-6.16								
65	-8.09								
62	-6.37								
55	-5.88								
65	-5.65								

as-synthesized		as-synthesized		as-synthesized		as-synthesized		as-synthesized	
Ag at.%	j_{mass} / A mg^{-1}	Ag at.%	j_{mass} / A mg^{-1}	Ag at.%	j_{mass} / A mg^{-1}	Ag at.%	j_{mass} / A mg^{-1}	Ag at.%	j_{mass} / A mg^{-1}
61	-8.23	62	-5.74	64	-8.25	65	-4.24	69	-6.56
64	-5.99	51	-3.10	62	-6.29	72	-5.03	51	-4.69
47	-6.26	60	-2.08	18	-3.13	65	-3.71	38	-5.97
58	-6.77	59	-4.76	63	-10.87	58	-6.46	63	-8.56
78	-22.01	48	-7.68	63	-8.21	55	-5.92	66	-8.86
41	-7.71	71	-2.08	42	-6.29	60	-4.23	69	-9.31
50	-12.66	62	-4.97	67	-14.53	66	-5.84	64	-7.77
67	-5.31	69	-6.26	53	-5.87	61	-5.70	65	-6.87
61	-10.00	59	-8.97	60	-6.94	42	-6.64	63	-5.88
43	-7.84	48	-13.80	76	-3.13	68	-8.44	54	-11.20
62	-7.25	58	-5.43	65	-3.29	58	-7.07	65	-2.45
62	-10.48	54	-7.83	66	-4.02	47	-3.89	76	-6.12
68	-6.32	61	-2.91	64	-4.53	58	-5.16	56	-6.98
60	-5.11	70	-3.66	65	-6.64	66	-8.42	64	-6.66
61	-7.59	64	-4.79	62	-4.40	46	-4.41	63	-7.89
64	-4.29	66	-17.54	75	-4.32	54	-5.85	53	-7.26
33	-7.69	67	-6.39	63	-6.19	50	-4.10	65	-5.15
60	-5.63	54	-8.51	62	-5.77	60	-3.25	68	-0.78
40	-4.86	60	-4.14	69	-4.99	63	-7.13	58	-11.23
24	-7.01	64	-5.48	68	-7.94	73	-1.45	49	-9.00
47	-12.99	56	-9.03	59	-5.98	75	-3.44	59	-4.66
35	-10.26	46	-7.59	62	-5.76	71	-3.94	58	-3.80
63	-11.73	43	-11.06	61	-10.31	69	-5.87	59	-5.32
45	-5.91	57	-7.19	63	-8.30	65	-7.01	66	-5.77
67	-6.29	46	-4.75	43	-5.46	63	-10.03	72	-6.50
61	-6.61	100	-12.61	52	-8.35	49	-5.33	56	-4.54
66	-7.32	47	-4.03	67	-4.78	69	-10.32	73	-5.56
48	-6.64	74	-2.79	66	-5.23	41	-3.66	49	-6.24
57	-10.06	69	-5.37	81	-11.75	88	-10.38	77	-12.60
63	-6.68	70	-7.34	51	-5.33	56	-6.18	52	-3.56
57	-6.07	55	-5.89	53	-5.70	54	-7.52	39	-10.43
50	-10.41	73	-4.11	63	-7.24	69	-7.58	55	-10.43
61	-5.67	53	-5.12	56	-3.77	52	-6.32	50	-5.56
65	-6.78	71	-5.23	57	-5.36	64	-4.93	43	-7.65
69	-13.70	74	-2.50	37	-6.85	52	-7.22	72	-2.11
100	-6.52	72	-4.00	61	-5.99	58	-14.19	70	-4.01
61	-8.32	67	-2.30	26	-10.70	50	-2.87	50	-6.64
63	-7.19	74	-8.38	78	-4.57	57	-7.03	67	-5.99
71	-23.07	69	-7.20	79	-4.95	74	-6.45	63	-8.33
48	-5.88	54	-7.76	75	-9.63	75	-1.49	66	-7.53
61	-6.50	62	-8.11	70	-2.37	67	-5.04	43	-5.06
58	-4.68	61	-5.51	68	-6.61	64	-6.64	19	-4.22

**as-synthesized**		**as-synthesized**		**as-synthesized**		**as-synthesized**		**as-synthesized**	
**Ag at.%**	**j_{mass} / A mg^{-1}**	**Ag at.%**	**j_{mass} / A mg^{-1}**	**Ag at.%**	**j_{mass} / A mg^{-1}**	**Ag at.%**	**j_{mass} / A mg^{-1}**	**Ag at.%**	**j_{mass} / A mg^{-1}**
56	-2.56	74	-4.26	63	-10.09	66	-3.99	36	-2.32
56	-4.14	67	-4.57	64	-6.41	32	-6.55	86	-2.69
56	-6.64	71	-6.25	40	-5.59	74	-6.55	75	-4.95
56	-4.95	60	-6.50	31	-3.64	81	-1.57	68	-2.35
75	-5.04	43	-6.06	68	-10.12	72	-4.33	74	-2.71
75	-5.51	57	-7.66	74	-5.95	77	-1.62	80	-1.19
55	-6.16	62	-5.03	81	-4.70	72	-1.56	64	-2.11
65	-7.16	72	-7.38	83	-4.95	68	-4.17	54	-2.35
56	-4.03	47	-8.25	83	-1.43	75	-2.13	54	-5.02
62	-4.12	56	-5.84	78	-1.33	68	-3.35	56	-5.32
77	-5.11	46	-6.07	76	-1.64	58	-5.43	64	-3.39
76	-5.59	49	-6.92	72	-5.56	66	-2.11	45	-4.33
66	-4.76	59	-3.43	64	-4.30	49	-8.39	41	-4.34
79	-5.99	69	-3.94	68	-3.27	51	-9.43	54	-5.80
54	-5.41	77	-1.86	65	-2.29	54	-4.96	61	-7.28
63	-10.21	69	-2.87	71	-3.14	67	-5.81	50	-7.48
54	-3.96	67	-3.19	63	-6.15	61	-7.77	23	-9.81
49	-6.18	73	-4.53	62	-5.73	72	-8.01	64	-6.01
82	-0.79	58	-6.18	50	-4.55	83	-7.46	51	-6.62
78	-1.44	48	-7.58	62	-7.40	56	-12.74	59	-8.06
72	-2.91	54	-3.69	57	-13.14	30	-11.84	48	-6.18
75	-4.45	65	-8.72	46	-4.11	48	-5.84	61	-2.81
65	-4.13	62	-4.12	52	-7.06	56	-8.13	56	-3.00
68	-5.46	57	-6.35	52	-8.97	40	-7.40	36	-8.03

S4. Details on IL-STEM/EDX Analysis

S4.1. Dealloying of Ag_xAu_y Nanoparticles on BDD Electrode

For IL-STEM imaging, the Ag_xAu_y nanoparticles were electrochemically dealloyed on a BDD electrode using a 3-electrode setup as depicted in Figure S7a. To avoid possible complications caused by the platinum counter electrode, it was placed at a considerable distance (several cm) from the working electrode. This configuration was only used for IL-STEM/EDX, and Pt was never found in this analysis. The resulting dealloying CV curves (Figure S7b) show the two peaks A1 and A2 at the first cycle, while the faradaic current is much smaller than on the GC macroelectrode (Figure 1a). Besides the smaller electrode area, this would indicate that less material was deposited per unit area. However, the particle coverage was similar on BDD compared to GC (see Figure S4b and S8). After the first cycle, all following cycles are almost featureless. However, previous studies have shown that structural and compositional changes during dealloying can still occur when (almost) no peaks are visible in the CV.[13,14] This is evident from our results as well.

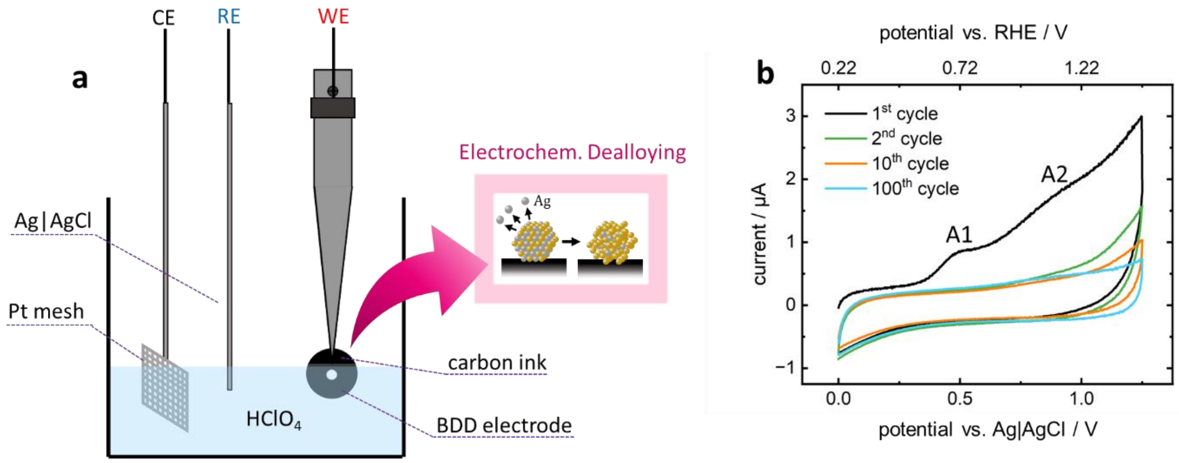

Figure S7. Electrochemical dealloying of $Ag_{60}Au_{40}$ on a BDD electrode for IL-STEM/EDX measurements. (a) Experimental setup for potentiodynamic dealloying in four steps using a metal tweezer to electrically contact the BDD electrode (WE) in a 1.0 M $HClO_4$ solution, as well as a leakless Ag|AgCl RE and a Pt mesh counter electrode (CE). (b) Selected CVs of electrochemical dealloying using a scan rate of 1 V s^{-1}.

S4.2. Coverage of As-Deposited Particles on BDD Electrode for IL-STEM/EDX

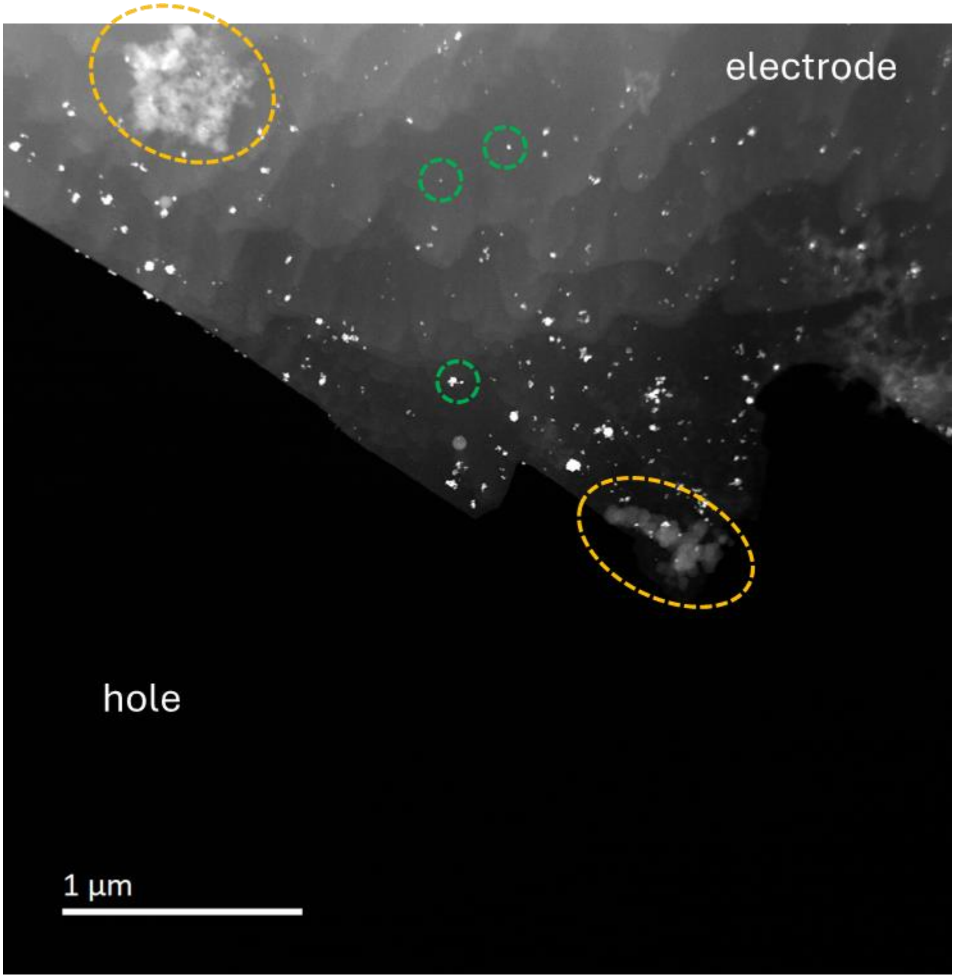

Figure S8. Low magnification dark-field STEM image showing the electron beam transparent edge of the central hole of the BDD electrode that was covered by electrosynthesized Ag_xAu_y entities (examples circled in green). The black area represents the hole (vacuum) in the STEM. Residues of the micellar polymer are circled in orange.

STEM images taken at low magnification, such as in Figure S8, demonstrate that the BDD electrode was covered by Ag_xAu_y entities (bright spots, examples circled in green). IL-STEM/EDX was performed in the electron-beam transparent area near the edge of the hole (black area) in the middle of the electrode. The cloud-shaped objects (circled in orange) that are less bright than the particles may be attributed to residues of the micellar polymer PS–P2VP.

S4.3. Morphological Changes to Ag_xAu_y Entities during Dealloying

The Ag_xAu_y particles formed by nucleation from reverse micelles on the BDD electrode were found to be mostly spherical or oval. The series of IL-STEM images in Figure S9 demonstrates that the centre of mass for the Ag_xAu_y entities is relatively consistent throughout the four-step dealloying treatment *i.e.* no significant movement is noted. Before dealloying, particle aggregates consisting of two or more particles in close contact with each other were found as well as primary nanoparticles.

In the first image of Figure S9, we counted 53 primary nanoparticles and 52 aggregates, noting that in limited cases it was difficult to assign unambiguously. To generate size distributions at each dealloying state (Figure 4b), the diameter of 70 primary nanoparticles was measured with the software ImageJ. When comparing the IL-STEM images in Figure S9 from "before dealloying" and after 100.5 cycles of dealloying, the number of identifiable aggregates decreased to only 17, while the number of primary particles (isolated particles that appear circular or oval) increased to 87. Higher-magnification imaging was necessary to identify the processes behind the observed changes.

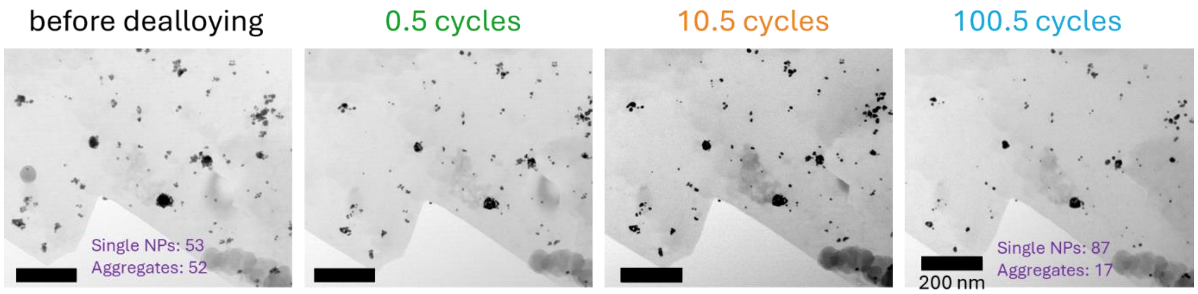

Figure S9. Low magnification STEM images at four different dealloying states. As-synthesized particles (before dealloying) include some aggregates (entities that could not be identified as individual nanoparticles but consist of two or more particles in close contact; about 50 % of the objects counted in left image). The number of aggregates decreased upon dealloying and made up only ~ 16 % of the objects counted in the area of the image taken after 100.5 cycles of dealloying, while the number of primary particles increased.

Figure S10 shows one example out of 20 high-magnification image series, demonstrating different events during dealloying. Within the two aggregates (marked by red and yellow circles), some of the Ag_xAu_y particles merged, forming objects that could not be distinguished from aggregated primary particles. This process is called particle "coalescence".[16] The aggregate marked by the red circle, which consisted of four primary particles in the as-synthesized state, turned into one single, spherical particle after 100.5 dealloying cycles. The aggregates coalesced throughout the treatment, while the 90 cycles between the 3rd and 4th step caused the strongest morphological difference.

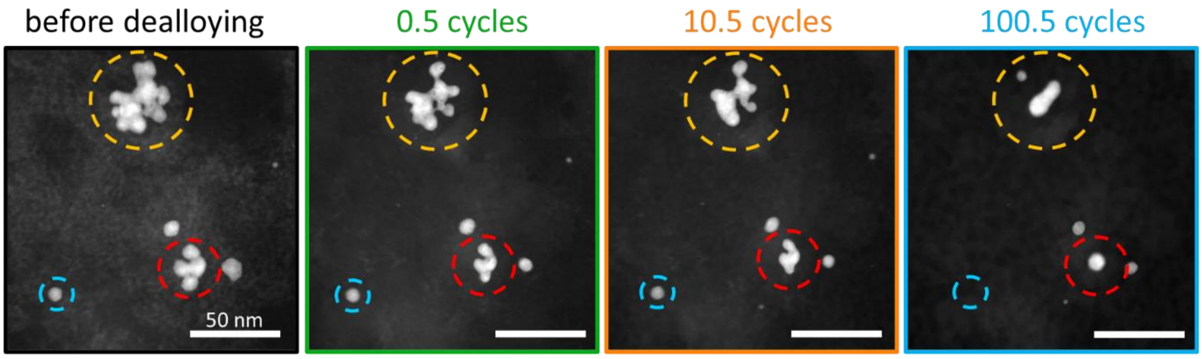

Figure S10. High-magnification IL-STEM imaging series showing different events during dealloying of Ag_xAu_y nanoparticles on BDD: red: coalescence of particle aggregate, yellow: coalescence and particle disintegration, blue: particle disappearance.

Interestingly, one of the primary particles in the yellow-marked aggregate, separated from the aggregate. Similar events of dealloying-driven detachment of primary particles from aggregates were observed in other locations on the electrode but were less common than coalescence. Furthermore, the primary particle marked in blue disappeared over the last 90 cycles of the dealloying treatment. This complete dissolution (or possible detachment) was observed for 7 out of 25 nanoparticles, especially after the last dealloying step that contained the highest number of cycles. Figure S10 shows how the decreasing ratio between aggregates and primary particles – observed in the low-magnification imaging in Figure S9 – is mainly a result of particle coalescence, where particle dissolution and disintegration from aggregates also play a role. In total, 2 aggregates and 3 primary particles transform into 1 aggregate and 4 primary particles.

S4.4. Calculation of Theoretical Particle Size from EDX Composition

This calculation was done to determine if the shrinking of the particles during dealloying can be attributed to the loss in Ag content alone or if any Au was expected to be dissolved. Based on the average Ag content from EDX data and the average diameter of the as-synthesized particles from STEM, the theoretical average particle diameter $\bar{d}_{\mathrm{theo}}$ was calculated for the case that only Ag and no Au was dissolved. The calculation was made under the assumptions that the particles had a spherical shape without any pores or surface roughness, and constant density throughout all dealloying steps.

If no Au is dissolved during dealloying, the average number of Au atoms per nanoparticle should not change and be equal to the average number of Au atoms in the as-synthesized nanoparticles. The average diameter $\bar{d}_0$ and average Ag content $\bar{p}_{\mathrm{Ag,0}}$ and thus, average Au content $\bar{p}_{\mathrm{Au,0}} = 1 - \bar{p}_{\mathrm{Ag,0}}$, are known from the IL-STEM/EDX analysis (Figure 4b,e). Assuming the volume $\bar{V}_0$ to be that of a sphere with diameter $\bar{d}_0$, the number of Au atoms in the average as-synthesized particle can thus be calculated using the average mole numbers $\bar{n}_{\mathrm{Au,0}}$, $\bar{n}_{\mathrm{Ag,0}}$, the molar masses M_{Au}, M_{Ag}, and the density of the Ag_xAu_y alloy ρ_{AgAu}:

$$\bar{V}_0 = \frac{\bar{n}_{\mathrm{Au,0}} M_{\mathrm{Au}} + \bar{n}_{\mathrm{Ag,0}} M_{\mathrm{Ag}}}{\rho_{\mathrm{AgAu}}} = \frac{4}{3}\pi\left(\frac{\bar{d}_0}{2}\right)^3 \quad (5).$$

The mole number $\bar{n}_{\mathrm{Ag}}$ can be replaced using Equation (3):

$$\frac{1}{\rho_{\mathrm{AgAu}}}\left(\bar{n}_{\mathrm{Au,0}} M_{\mathrm{Au}} + \frac{\bar{p}_{\mathrm{Ag,0}}}{1-\bar{p}_{\mathrm{Ag,0}}}\bar{n}_{\mathrm{Au,0}} M_{\mathrm{Ag}}\right) = \frac{4}{3}\pi\left(\frac{\bar{d}_0}{2}\right)^3 \quad (6).$$

Equation (6) can then be rearranged to yield $\bar{n}_{\mathrm{Au,0}}$:

$$\bar{n}_{\mathrm{Au,0}} = \frac{\frac{4}{3}\pi \cdot \left(\frac{\bar{d}_0}{2}\right)^3 \rho_{\mathrm{AgAu}}}{M_{\mathrm{Au}} + \frac{\bar{p}_{\mathrm{Ag,0}}}{1-\bar{p}_{\mathrm{Ag,0}}} M_{\mathrm{Ag}}} \quad (7).$$

The number of Au atoms is given by $\bar{N}_{\mathrm{Au,0}} = \bar{n}_{\mathrm{Au,0}} \cdot N_{\mathrm{A}}$ where N_{A} is the Avogadro constant. Using $\bar{d}_0 \approx 11\ \mathrm{nm}$, $\bar{p}_{\mathrm{Ag,0}} \approx 0.6$, $M_{\mathrm{Au}} \approx 196.97\ \mathrm{g\ mol^{-1}}$,[7] $M_{\mathrm{Ag}} \approx 107.87\ \mathrm{g\ mol^{-1}}$,[8] $N_{\mathrm{A}} \approx 6.022 \cdot 10^{23}\ \mathrm{mol^{-1}}$,[17] and $\rho_{\mathrm{AgAu}} \approx 13.0\ \mathrm{g\ cm^{-3}}$ as a measured value for the $Ag_{60}Au_{40}$ alloy,[18] $\bar{N}_{\mathrm{Au,0}}$ was found to be approximately 15207. If we assume that this number stays constant over the dealloying treatment, the theoretical diameter $\bar{d}_{\mathrm{theo}}$ can be calculated by replacing $\bar{d}_0$ by $\bar{d}_{\mathrm{theo}}$ in Equation (6) and rearranging the equation for $\bar{d}_{\mathrm{theo}}$:

$$\bar{d}_{\mathrm{theo}} = 2\left(\frac{3}{4\pi}\frac{1}{\rho_{\mathrm{AgAu}}}\left(\frac{15207}{N_{\mathrm{A}}} M_{\mathrm{Au}} + \frac{\bar{p}_{\mathrm{Ag,0}}}{1-\bar{p}_{\mathrm{Ag,0}}}\frac{15207}{N_{\mathrm{A}}} M_{\mathrm{Ag}}\right)\right)^{1/3} \quad (8).$$

Here, the value for ρ_{AgAu} must be adapted according to the altered alloy composition after each dealloying step. For a rough estimation, the average values of the Ag content distributions (Figure 4e) were taken to read the respective values from the density curve measured by Kraut and Stern.[18] Based on these values, $\bar{d}_{\mathrm{theo}}$ was calculated as ~ 10 nm after 0.5 cycles, ~ 9 nm after 10.5 cycles, and ~ 9 nm after 100.5 cycles.

S4.5. Example for EDX Analysis

IL-EDX mapping of Ag and Au were performed at multiple Ag_xAu_y entities in each dealloying state. One representative example is presented in Figure S11. The homogeneous distribution of Ag and Au inside the 2D representation of the particle at every dealloying state confirms that the alloy nature of the particle does not change as a result of Ag dissolution and Au reorganization. No core-shell formation or other phase separations were observed in any of the EDX-mapped particles. Furthermore, the spherical morphology was retained.

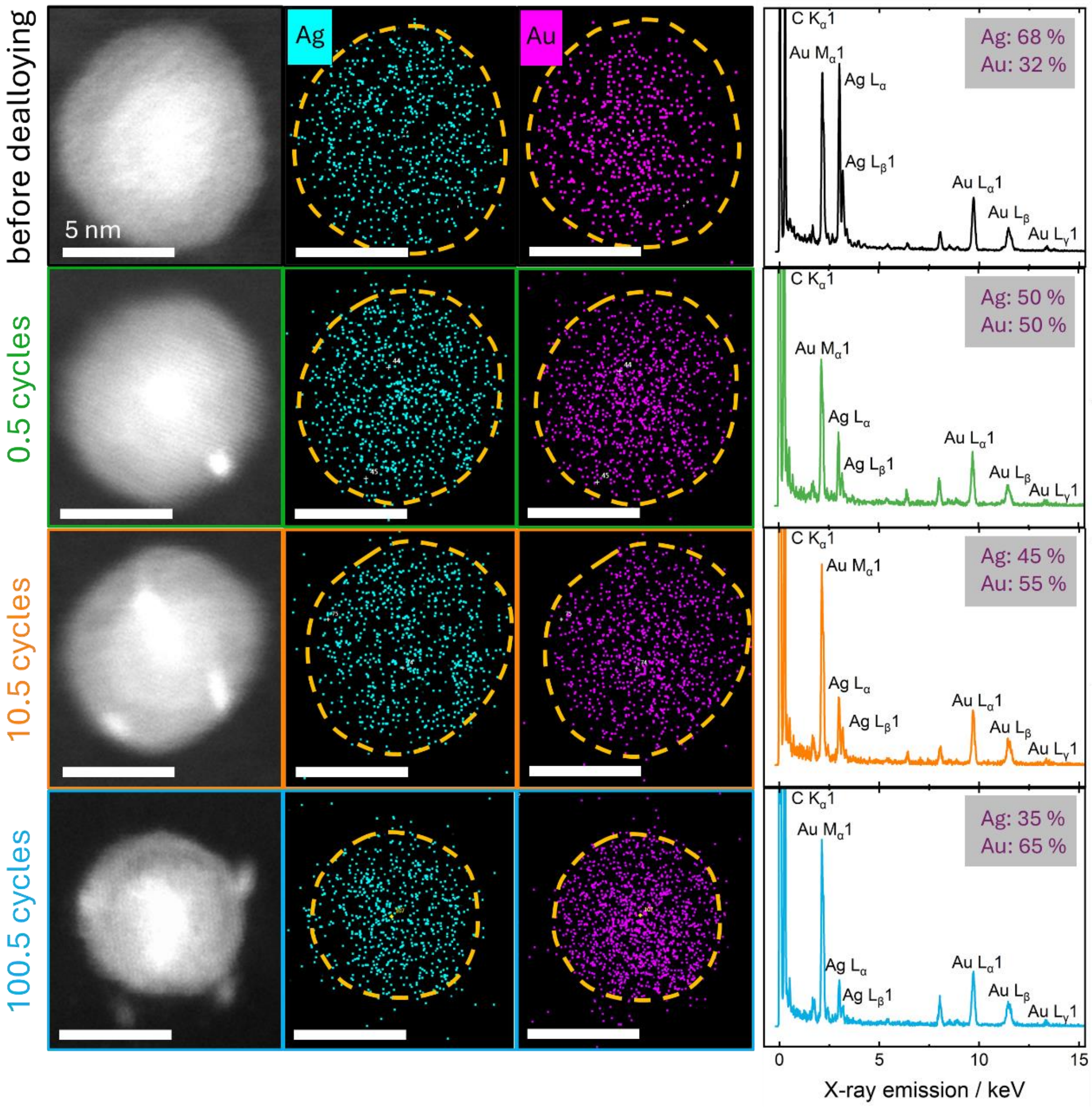

Figure S11. IL-STEM images and EDX mapping (with particle outlines from the STEM images) of the same primary Ag_xAu_y nanoparticle at four states of dealloying (after 0, 0.5, 10.5 and 100.5 cycles) with respective EDX spectra, zoomed into the Ag and Au peaks (labelling of the emission wavelengths according to *refs* [19,20] and intensity axes were normalized to the height of the Au $L_\alpha 1$ peak). Quantification of Ag and Au atomic content by AZtec (Oxford Instruments). Note: The bright spots in the dark-field images of the 10.5 and 100.5 cycles state were caused by carbon deposition during point-EDX measurements.

S4.6. Changes in Ag Content and Diameter over Dealloying

As an additional representation, the data presented in Figure 5 is also given in Table S3.

Table S3. Combined data of relative change in Ag content and diameter from IL-STEM/EDX (data presented in Figure 5) of as-synthesized and dealloyed primary particles and aggregates (only the diameters of primary particles were taken). Each row in the table refers to an individual entity (primary particle or aggregate) in the identical location measurement. For some primary particles, no entries were made from a certain dealloying step onwards, which means that the particle disappeared after this dealloying step.

As-synthesized Ag content / at.%	As-synthesized diameter / nm	Ag content change after 0.5 cycles / %	Diameter change after 0.5 cycles / %	Ag content change after 10.5 cycles / %	Diameter change after 10.5 cycles / %	Ag content change after 100.5 cycles / %	Diameter change after 100.5 cycles / %
68		-53	--	-67	--	-76	--
71		-47	--	-73	--	-74	--
63		-43	--	-49	--	-60	--
65		--	--	--	--	--	--
71		-57	--	-65	--	-75	--
64		-40	--	-47	--	--	--
58		-33	--	-41	--	-49	--
67		-51	--	-60	--	-73	--
63		-60	--	-79	--	-74	--
70		--	--	--	--	--	--
70	29.4	-62	-18.0	-64	-23.8	-75	-27.3
58	9.1	-13	-0.1	-21	-1.9	-39	-25.0
61		-14	--	-23	--	-51	--
68		-27	--	-28	--	-58	--
43	5.9	-58	-3.8	-73	-16.2	--	-51.9
60	11.0	-50	-13.6	-69	-21.5	-73	-43.0
61	11.1	-40	-12.2	-56	-15.9	--	-100.0
43	5.1	--	-32.2	--	-47.4	--	-100.0
65	13.5	-30	-18.0	-57	-23.3	-56	-46.0
43	6.3	-56	-24.1	-59	-24.7	-98	-64.1
66	9.0	--	-16.7	-65	-19.2	-92	-76.4
60	8.3	-16	-9.7	-21	-14.0	-94	-100.0
60	8.6	-54	-17.8	-58	-18.0	-90	-51.4
48	9.0	-44	-13.1	-41	-33.0	--	-100.0
56	9.0	-34	-1.7	-27	46.6	-60	38.4
57	9.3	-25	-1.2	-34	--	--	--
59	5.1	--	-100.0	--	-100.0	--	-100.0
59	7.9	-20	0.1	-37	-9.3	--	-100.0
42	7.3	--	-49.4	--	-100.0	--	-100.0

References

(1) Michael Faraday. VI. Experimental researches in electricity.-Seventh Series. *Phil. Trans. R. Soc.* **1834**, *124*, 77–122. DOI: 10.1098/rstl.1834.0008.

(2) Strong, F. C. Faraday's laws in one equation. *J. Chem. Educ.* **1961**, *38* (2), 98. DOI: 10.1021/ed038p98.

(3) Saw, E. N.; Grasmik, V.; Rurainsky, C.; Epple, M.; Tschulik, K. Electrochemistry at single bimetallic nanoparticles - using nano impacts for sizing and compositional analysis of individual AgAu alloy nanoparticles. *Faraday Discuss.* **2016**, *193*, 327–338. DOI: 10.1039/C6FD00112B.

(4) Gallego, J. H.; Castellano, C. E.; Calandra, A. J.; Arvia, A. J. The electrochemistry of gold in acid aqueous solutions containing chloride ions. *J. Electroanal. Chem.* **1975**, *66* (3), 207–230. DOI: 10.1016/S0022-0728(75)80004-0.

(5) Kelsall, G. H.; Welham, N. J.; Diaz, M. A. Thermodynamics of Cl-H2O, Br-H2O, I-H2O, Au-Cl-H2O,Au-Br-H2O and Au-I-H2O systems at 298 K. *J. Electroanal. Chem.* **1993**, *361*, 13–24.

(6) National Institute of Standards and Technology. *CODATA Recommended Values:Faraday Constant*. https://physics.nist.gov/cgi-bin/cuu/Value?f (accessed 2024-12-14).

(7) IUPAC Commission on Isotopic Abundances and Atomic Weights. Standard Atomic Weights of 14 Chemical Elements Revised. *Chemistry International* **2018**, *40* (4), 23–24. DOI: 10.1515/ci-2018-0409.

(8) CIAAW - Commission on Isotopic Abundances and Atomic Weights. *Standard Atomic Weights*. https://www.ciaaw.org/atomic-weights.htm (accessed 2024-12-14).

(9) Lu, X.; Yu, T.; Wang, H.; Luo, R.; Liu, P.; Yuan, S.; Qian, L. Self-Supported Nanoporous Gold with Gradient Tin Oxide for Sustainable and Efficient Hydrogen Evolution in Neutral Media. *JRM* **2020**, *8* (2), 133–151. DOI: 10.32604/jrm.2020.08650.

(10) Connor, P.; Schuch, J.; Kaiser, B.; Jaegermann, W. The Determination of Electrochemical Active Surface Area and Specific Capacity Revisited for the System MnO x as an Oxygen Evolution Catalyst. *Z. Phys. Chem.* **2020**, *234* (5), 979–994. DOI: 10.1515/zpch-2019-1514.

(11) *Warwick Electrochemical Scanning Probe Microscopy (WEC-SPM) platform*. https://warwick.ac.uk/electrochemistry/wec-spm (accessed 2024-09-05).

(12) Wahab, O. J.; Kang, M.; Daviddi, E.; Walker, M.; Unwin, P. R. Screening Surface Structure-Electrochemical Activity Relationships of Copper Electrodes under CO2 Electroreduction Conditions. *ACS Catal.* **2022**, *12* (11), 6578–6588. DOI: 10.1021/acscatal.2c01650.

(13) Rurainsky, C.; Manjón, A. G.; Hiege, F.; Chen, Y.-T.; Scheu, C.; Tschulik, K. Electrochemical dealloying as a tool to tune the porosity, composition and catalytic activity of nanomaterials. *J. Mater. Chem. A* **2020**, *8* (37), 19405–19413. DOI: 10.1039/D0TA04880A.

(14) Rurainsky, C.; Nettler, D.-R.; Pahl, T.; Just, A.; Cignoni, P.; Kanokkanchana, K.; Tschulik, K. Electrochemical dealloying in a magnetic field – Tapping the potential for catalyst and material design. *Electrochim. Acta* **2022**, *426*, 140807. DOI: 10.1016/j.electacta.2022.140807.

(15) Schroeder Rossi, T.; Papaderakis, A. A.; Jaugstetter, M.; Jlailati, Z.; Knoke, M.; Hosseini, P.; Cignoni, P.; Yang, F.; Gerwin, M.; Trost, O.; Spallek, M.; Ortega, E.; Roldan Cuenya, B.; Crans, D. C.; Levinger, N. E.; Tschulik, K. Bimetallic Ag–Au nanoparticles from nanoconfinement: adjusting properties by electrochemical synthesis. *J. Mater. Chem. A* **2025**, *13*, 24014–24027. DOI: 10.1039/D5TA01833A.

(16) José-Yacaman, M.; Gutierrez-Wing, C.; Miki, M.; Yang, D.-Q.; Piyakis, K. N.; Sacher, E. Surface diffusion and coalescence of mobile metal nanoparticles. *J. Phys. Chem. B* **2005**, *109* (19), 9703–9711. DOI: 10.1021/jp0509459.

(17) National Institute of Standards and Technology. *CODATA Recommended Values:Avogadro constant*. https://physics.nist.gov/cgi-bin/cuu/Value?na (accessed 2024-12-14).

(18) Kraut, J. C.; Stern, W. B. The density of gold-silver-copper alloys and its calculation from the chemical composition. *Gold Bull* **2000**, *33* (2), 52–55. DOI: 10.1007/BF03216580.

(19) J. A. Bearden. *X-Ray Wavelengths*; Review of Modern Physics, 1967. pp. 86-99.

(20) Krause, M. O.; Oliver, J. H. Natural widths of atomic K and L levels, K α X-ray lines and several KLL Auger lines. *J. Phys. Chem. Ref. Data* **1979**, *8* (2), 329–338. DOI: 10.1063/1.555595.
